# Reviewing the User-Centered Design Process for a Comprehensive Gastroesophageal Reflux Disease (GERD) App

**DOI:** 10.3390/ijerph19031128

**Published:** 2022-01-20

**Authors:** Min Ji Kim, Sarah Schroeder, Shuan Chan, Kyle Hickerson, Yi-Ching Lee

**Affiliations:** Human Factors and Applied Cognition, Psychology Department, George Mason University, Fairfax, VA 22030, USA; sarah.lenore.schroeder@gmail.com (S.S.); schan20@gmu.edu (S.C.); khicker@gmu.edu (K.H.); ylee65@gmu.edu (Y.-C.L.)

**Keywords:** human factors, mobile apps, digital health, mHealth, design, usability, user testing, behavior change, health psychology, health interventions

## Abstract

The objective of this study was to design a user-centered mobile health (mHealth) application for individuals with gastroesophageal reflux disease (GERD) and evaluate its design features and effectiveness for use by doctors. Prior to designing, our team undertook a discovery process that involved creating personas, conducting a competitor analysis and heuristic evaluation of existing apps, along with interviews with acid reflux patients. Then, we created a low-fidelity prototype, which was revised on the basis of several rounds of user testing. During the design phase, each round of user testing included a mix of surveys, concurrent think-alouds, and interviews to gather user feedback on the prototypes. Lastly, an evaluation phase consisting of gathering feedback on the user-centered design approach from user experience experts and medical doctors specialized in GERD was conducted. Overall, the final GERD app includes important features for tracking symptoms and triggers, analytics, data export, and community information, while promoting individualization, accessibility, and usability. The documentation of the design process of this app serves as a reference point for future medical app developers as it followed an empirically supported user-centered design strategy and resulted in an app which received positive feedback from users and human factors experts. We also intend to share some of the limitations due to the constrained resources, as well as potential ways to strengthen the design process for mHealth applications.

## 1. Introduction

Gastroesophageal reflux disease (GERD) is a medical condition related to reflux of stomach contents into the esophagus [1,2]. GERD is often associated with heartburn [3] but may manifest via atypical symptoms, such as chronic cough, cardiac arrhythmias, or sleep apnea [1]. Both the causes and course of GERD are complex and vary from patient to patient, with diverse clinical histories, endoscopic test results, ambulatory reflux text results, and esophageal characteristics interacting to produce more than 50 phenotypic classifications of GERD that may respond optimally to unique treatment protocols [2].

Global prevalence of GERD in adults ranges from about 2.5–7.8% in East Asia to about 18.1–27.8% in North America [4]. In general, advanced age is associated with an increase in GERD risk. One analysis, though, indicates that the overall proportion of GERD patients between the ages of 30 and 39 increased significantly between 2006 and 2016 [4], perhaps due to increased exposure across the lifespan to GERD risk factors.

Like advanced age, family history plays an important role in determining an individual’s GERD risk [4]. In addition, factors enmeshed with behavioral patterns—such as anxiety, obesity, and smoking—also have been linked to GERD in the empirical literature [1,5,6]. Observational evidence suggests that a worldwide increase in obesity has fueled an increase in GERD symptoms [1]. Some evidence also points to a worldwide decrease in infections caused by *Helicobacter pylori* bacteria as having potentially contributed to rising numbers of GERD cases because under certain circumstances *h. pylori* may exert a protective effect against GERD; however, more research is needed to fully understand the relationship between these two conditions [7]. 

GERD exacts a serious toll on both individual patients and society as whole. Common consequences of GERD include diminished quality of life, diminished productivity, and poor sleep quality [3,4,8]. GERD is rarely fatal by itself; nevertheless, it can lead to painful inflammation of the esophagus, peptic strictures, esophageal lesions, and an increased risk of esophageal cancer [6]. Furthermore, GERD diagnostics, treatments, surgeries, and drugs are expensive; In 2009 alone, GERD patients in the United States needed more than 8.8 million doctor’s visits and 65,000 hospitalizations and spent USD 12.3 billion on diagnostic upper endoscopies [1,9].

Traditionally, antacid medications and lifestyle modifications are considered first-line treatments for GERD. When these first-line treatments fail, surgery may become necessary to control GERD [3]. In addition to being costly, using first-line acid reflux drugs for a long time may lead to adverse effects, including increased acid reflux symptoms upon withdrawal, an increased susceptibility to infections, and an increased risk of bone fractures [3]. On the other hand, in some cases lifestyle changes—including weight loss, diet changes, and eliminating tobacco—may improve GERD symptoms at a low cost with few side effects [3,10,11]. 

It is a standard practice to suggest weight loss to obese GERD patients and to suggest smoking cessation [3,6]. Patients with nocturnal GERD often benefit from sleeping with their bed elevated and avoiding large meals right before bedtime [6,8]. Individual patients also may find it helpful to avoid certain foods which exacerbate or trigger their GERD symptoms [8,11]. In addition, there is some evidence that more general diet recommendations, such as following a traditional Mediterranean diet, increasing fiber intake, reducing meal size, and reducing fat and simple sugar intake, may be useful in controlling GERD [10].

Prior empirical research demonstrates that mobile phone applications (apps) and digital assistants can improve outcomes and everyday life for patients with numerous chronic health conditions, such as diabetes, asthma, chronic pain, cancer, and Parkinson’s disease. These mobile healthcare (mHealth) apps work by facilitating patient support and education; addressing behaviors, including medication and diet adherence; and monitoring key health measures (e.g., blood glucose for people with diabetes) [12,13,14,15,16]. mHealth can also be useful for promoting desirable knowledge and behaviors in healthy individuals—for example, by harnessing gamification to teach the mothers of young children about oral health and hygiene [17]. Many mHealth apps promote healthy lifestyle changes, such as increasing physical activity, eating a healthy diet, smoking cessation, and moderating alcohol intake [18].

Despite this promise, a review of recent literature suggests that the field of mHealth must undergo numerous transformations before reaching its full potential to improve patient care. These transformations include increasing the number of randomized control trials that compare mHealth interventions to current best practices; studying individual mHealth interventions over longer timeframes; conducting further studies to identify the conditions and features that allow mHealth apps to work best; implementing formal regulatory oversight to help patients distinguish high-quality apps from poor-quality ones; and improving privacy and security features of mHealth apps [13,15,17]. In achieving these transformative goals, researchers have suggested that studies evaluating mHealth apps must rely on precise, standardized measurements, including measures related to usability [19,20].

### Aim and Objectives

In this paper, we aim to describe a user-centered research and design process for a novel mobile application created to help GERD patients monitor and manage their condition. The specific objectives of this paper are to:

1. Document an iterative user-centered design and evaluation of an mHealth app;
2. Document challenges and lessons learned related to the user-centered design of mHealth apps.

Our prototype, titled “GerdHelper,” was initially designed as part of the 2021 Mobile Health Applications for Consumers Design Competition [21], sponsored by the Human Factors and Ergonomics Society. “GerdHelper” was built to address symptoms of GERD. 

We entered the contest in December 2020 and submitted our final entry on 19 February 2021 in accordance with contest timelines. 

We chose to design an app related to GERD management in consideration of our understanding of GERD as a chronic disease whose treatment can depend on a variety of behaviors [3,10,11] (and our understanding that mHealth apps hold promise for improving the management of chronic diseases) [12,13,14,15,16].

Our team also based the decision to build an app addressing GERD on a competitive analysis of popular GERD apps available in the App Store, which showed that existing apps are difficult to use and navigate. We conducted a content analysis of these apps by reviewing how the apps addressed key features we had identified as important based on our research on GERD symptoms and treatments. We reviewed features such as tracking GERD symptoms, obtaining suggestions about lifestyle, exporting data about symptoms, and community information. We found that the competitor apps attempted to provide these features via features such as calendars, tips, and data export options, but none of the apps were able to provide all the features, and the provided features had room to be improved upon. Even the highest rated competitor app, the Acid Reflux Helper, did not have all the features, and had unintuitive and unaesthetic features. Our conclusion was supported by Bobian et al.’s 2017 [22] analysis of existing GERD apps, which showed that available GERD apps use prohibitively complicated language. In response, our team endeavored to build a better GERD app through four iterative stages that incorporated best practices in user-centered design, as well as an in-depth evaluation process. We aim to share lessons learned and strengthen the literature on the human-centered design of mHealth apps.

## 2. Materials and Methods

This study spanned a design phase and an evaluation phase. During the design phase, we used a variety of empirically supported methodologies to create and test our prototypes: We created personas, conducted a competitor analysis, completed a heuristic evaluation, created a prototype, conducted interviews, completed 2 rounds of user testing with think-alouds and semi-structured interviews, and, finally, consulted experts after we created the final prototype. Our three rounds of user testing—each followed by refinements of our prototype—represented multiple iterations of the design thinking process.

During the evaluation phase, we used formative methods to evaluate our app on the basis of feedback from the design competition judges, input from experts in GERD that was solicited via an online survey, and our own review of current literature. Our goal during the evaluation phase was to draw conclusions about the strengths and weaknesses of our app and share lessons learned with the broader scientific and medical community.

### 2.1. Goals for the Design Phase

**Figure 1:**
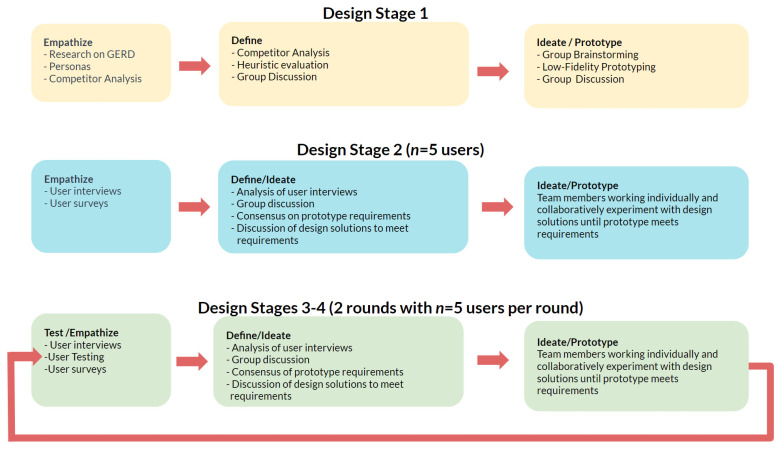
Rough map of the user-centered design thinking process behind GerdHelper, where design is iterative and design-thinking components are not strictly sequential or discrete.

Our team relied on the design thinking process for underlying guidance during the design phase, following the five-component structure—empathize, define, ideate, prototype, and test [23]—and focusing on what users need and how what we created would respond to our users’ needs [24]. As is standard in user-centered research, these components were not always applied sequentially or discretely during the iterative design of the GerdHelper app. A map of the rough overlap between traditional design-thinking components and our team’s four-stage design process is shown in Figure 1.

This research was approved by the George Mason University Institutional Review Board (IRB # 1708698-2). All user testing was conducted online through Zoom, and the iterative design process was broken down into four design stages. During Stage 1, we created personas on the basis of two research methods geared towards empathizing with likely users: (1) review of literature on GERD symptoms, on patient characteristics, and on effective treatment techniques; and (2) a competitor analysis which shed light upon likely users’ mental model of available medical applications. Given the time and monetary constraints governing this project, these research techniques were chosen over more time consuming means of empathizing with users, such as focus groups and observational studies. Combined with a heuristic evaluation of currently available apps, our literature review and competitor analysis allowed us to define key app features ahead of a series of team meetings used for ideation. We then created one low-fidelity prototype that improved upon existing GERD apps, as well as a medium-fidelity prototype. In Stage 2, we interviewed five participants with acid reflux to empathize with them and gather their input regarding desirable features, which we incorporated into the preexisting second, medium-fidelity iteration of the prototype. Design Stage 3 involved five users completing tasks in the app and completing a concurrent think-aloud. Afterwards, we interviewed the five users about app features and navigation, and we incorporated user feedback into the third iteration of the prototype. Design Stage 4 repeated the process followed in Stage 3 to inform the final version of the prototype. Our design thinking process followed a similar course during stages 2–3 whereby we gathered user feedback through user interviews and/or usability testing and then met as a group—sometimes more than once—to review user feedback, define required improvements on the basis of that feedback, ideate practical solutions, and then build them into our prototype before another round of user testing. The design process applied in building the GerdHelper App is outlined in Figure 1.

#### 2.1.1. Stage 1 Design Methods: Personas, Competitor Analysis, and Heuristic Evaluation

The primary objective of Stage 1 was to create the first iteration of the prototype. This task was achieved through personas, a competitor analysis, and heuristic evaluation. Details about Stage 1 and the resulting features of the app are described in Supplementary A and summarized here (See Figures S1 and S2 and Tables S1–S3): 

Personas are fictional representations that facilitate empathy for target users [25]. They communicate information, such as the background and goals of users, with the goal of informing a user-centered design process. Our team used three realistic personas, each representing a different facet of our broad target user audience, to act as a frame of reference to our user group and kickstart our design process:

- Smoker Samuel, 48, wanted to enjoy everyday activities by reducing GERD symptoms;
- Pregnant Patricia, 32, wanted to stay well-rested and improve the outcome of her pregnancy by reducing her level of GERD-related discomfort;
- Stressed-Out Sophia, 25, wanted to determine whether her symptoms could be attributed to GERD and learn more about how to manage GERD symptoms.

Full details of the three user personas can be found in Table S1. 

Once the needs, motivators, and goals of our potential users were outlined, we identified four main features that would meet user needs: (1) tracking acid symptoms, (2) analytics and suggestions about a healthier life, (3) exporting the data about symptoms, and (4) finding community information about acid reflux.

We made these four features the target of a competitor analysis. Competitor analysis is a method frequently used in product design where experts assess the strengths and weaknesses of existing competitor products. This method allows designers to empathize with users by understanding their mental model of existing products. Further, it helps researchers define key user needs and determine whether current products meet those needs, where current products fail to meet the needs, and how the to-be-created product can fill gaps in the market [26]. We chose three existing mobile apps for GERD/Acid reflux management: (1) Acid Reflux Diet Helper [27]; (2) mySymptoms Food Diary & Symptom Tracker [28]; and (3) Refluxlog [29]. These apps were chosen because they had features similar to the ones that we had determined we should include in our own app. For each app, we tested four key tasks, each of which was chosen for its importance to the four end-user needs identified as central to our app design:

- Task 1: track GERD symptoms;
- Task 2: provide suggestions about lifestyle;
- Task 3: export symptom data; and
- Task 4: view community information.

To further review important features of these competitor apps and inform our own definition of important app features, we conducted a heuristic evaluation. Heuristic evaluation is a method where usability experts evaluate a product to see if it meets established usability principles [30]. We evaluated our chosen competitor apps with four usability principles—effectiveness/errors, efficiency, satisfaction, and learnability [30,31,32]—and we split some of these principles to fine tune our evaluation. We divided error/effectiveness to two separate principles—effectiveness and forgiveness—to emphasize the degree to which the app allows the users to recover from mistakes easily. We also divided satisfaction into aesthetic satisfaction and satisfaction with the quality of the outcome to understand the degree to which the apps are satisfactory/unsatisfactory for aesthetic reasons or for its functions. Each of these heuristics were evaluated in the same four tasks that were used during competitor analysis.

In addition to conducting formal qualitative evaluations through personas, competitor analysis, and heuristic evaluation, Design Phase 1 involved a series of team meetings for the purpose of defining prototype requirements and ideating design solutions by which those requirements could be met. More information about specific design decisions made during Design Stage 1 are available in Section 3.1.1, as well as in Supplementary A.

#### 2.1.2. Stage 2 Design Methods: Semi-Structured Interviews

Empathizing with users and defining user needs—two key elements of the design thinking approach implemented in this study—require soliciting input from users early and often in the design process. In line with this goal, we interviewed potential users during Stage 2 of our design process. Interview questions used during Design Stage 2 are described in Supplementary B and outlined here.

A semi-structured interview is a research method that utilizes closed-ended and open-ended questions to probe participants about the hows and whys of their answers [33,34]. This method allows researchers to gain a more thorough understanding of the participant than would be possible via a survey while still keeping a level of objectiveness. We chose this method in order to allow participants to answer specific questions, but also to elaborate about their choices. The semi-structured interview method also allowed us to probe interesting points that the participants brought up.

In Design Stage 2, we interviewed five participants (2 female, 1 male, 2 non-binary) who suffer from acid reflux in the age range of 24 to 35 years. Due to the time limitations imposed by the 2021 Mobile Health Applications for Consumers Design Competition, we recruited a convenience sample from among our personal social circles using social media to find contacts who experienced acid reflux symptoms. 

The sessions were scheduled over Zoom, and each lasted approximately 45 min. Participants’ audio was recorded, so we could review feedback throughout the design process. A researcher asked participants about their acid reflux symptoms and what they would like to see in an app that manages acid reflux. Participants’ answers were documented, and during a series of meetings our team worked together to define updated prototype requirements and ideate practical design solutions that would meet those requirements. These discussions inform a testable prototype using the interactive prototyping software Figma [35]. These specific design decisions are described in Section 3.1.2, as well as in Supplementary B.

#### 2.1.3. Stage 3 Design Methods: User Testing and Iterative Prototyping

The objective of Stage 3 was to observe users interacting with our prototype and refine the prototype according to their performance and feedback. Additional details from Design Stage 3 can be found in Supplementary C.

During Design Stage 3, a user testing and interview process allowed our team to continue building empathy for users’ experience navigating the GerdHelper app and their impression of the app’s functionality and aesthetics.

First, we interviewed five users (3 females, 1 male, and 1 non-binary) in the age range of 24 to 35 years. We chose a sample size of 5 users because of evidence that testing an iterate prototype on five users will allow user researcher to detect most major usability problems [36]. Participants were recruited from among our personal contacts using social media. First, users were asked to complete a pre-interview survey that included informed consent and demographic questions. 

Next, we showed users the Stage 2 prototype on Figma and asked them to complete tasks related to entering symptoms, accessing a calendar, and identifying foods that they should avoid. While executing these tasks, users were asked to complete a concurrent think-aloud. Think-aloud is a method where users verbalize their thoughts as they complete a given task. This method is useful because it allows researchers to gain insight into why and where novice users struggle, and understand how expert users solve specific tasks [37]. We chose to conduct a concurrent think-loud to observe direct articulation of what the participants were thinking as they completed each task [37]. However, to gain more complete thoughts about the app and its features, we followed the think-aloud with a semi-structured interview.

Through the think-aloud, we aimed to achieve deeper insights into users’ expectations about and experiences of the app than could be obtained by observation of users’ actions alone [38]. Next, participants completed an additional set of tasks, without completing a concurrent think-aloud and while being observed. Participants’ computer screens, facial expressions, and audio were recorded. Afterwards, a researcher interviewed each participant about their perception of the app, and participants completed a brief online survey about their opinions on our prototype.

After completion of this empathetic interview, testing, and survey process, our team met, defined updated prototype requirements on the basis of user-testing and user feedback, ideated design solutions that would meet said requirements, and then finally refined our prototype in preparation for a final round of user testing. Specific details about design decisions made during Stage 3 are available in Section 3.1.3 and in Supplementary C.

#### 2.1.4. Design Stage 4: Additional User Testing and Iterative Prototyping

We conducted a final design stage for the purpose of further fine tuning our prototype on the basis of a continuing empathetic and user-centered iterative design process and specifically testing usefulness of changes to the symptom tracking and analysis features that were revised during the previous design stage. We repeated the interview and survey procedures used in Design Stage 3 during Design Stage 4. Our users (4 females, 1 male) ranged from 18 to 44 years old and were recruited from our personal contacts using electronic correspondence. Information gathered during Stage 4 surveys and interviews informed the final iteration of the app prototype after a series of requirements and ideations meetings similar to those that took place during earlier stages of the design process. Additional details about Design Stage 4 are described in Section 3.1.4 and in Supplementary D.

### 2.2. Methods Used for the Evaluation Phase

At the conclusion of the 2021 Mobile Health Applications for Consumers Design Competition, our team conducted a qualitative evaluation on the basis of feedback from competition judges, comments from GERD experts, and our own review of academic literature on both GERD and mHealth apps.

#### 2.2.1. Feedback from the Competition Judges

Competition judges provided written feedback on our submission during the first round of competition, after which our team advanced to the second round of competition, where we presented our prototype to a three-judge panel of human factors experts during the 2021 International Symposium on Human Factors and Ergonomics in Healthcare. We then solicited feedback via email from the three-judge panel and had a follow-up conference call with one of the judges. 

#### 2.2.2. Feedback from GERD Experts

After the competition, we created a 17-question survey and solicited feedback from medical doctors specialized on GERD. Survey is a method where researchers can gather information from a sample size through a low-cost transmission [39,40]. This method is beneficial for its low cost, ease of response by users, and automatic transmission and response [40]. We chose to email a web-based survey to the experts as we recognized that the experts are busy and unlikely to be able to make time for a full semi-structured interview. 

Our survey was administered via the platform Qualtrics and included a link to our Figma prototype. We collected basic demographic information about each expert’s professional background and experience studying and/or treating GERD. We also asked expert respondents for open-ended feedback about behavioral aspects of GERD, the use of apps in treating GERD, and feedback regarding the usefulness of our specific app. Survey questions were not formally validated because the survey style was designed to resemble a semi-structured interview. 

Experts were identified through a Google Scholar search of recent peer-reviewed literature on GERD, as well as a Google search of primary care and gastroenterologists practicing in the Washington, DC metro area. We emailed a link to our survey to a total of 25 experts. One reminder email was also sent to each expert. Two experts submitted answers to all 17 questions. Seven respondents began to complete the survey questions but stopped before providing any open-ended input into behavioral aspects of GERD treatment or feedback about GerdHelper. The contents of this 17-question survey appear in Supplementary E.

#### 2.2.3. Evaluation Based on Previous Literature

In addition, we evaluated our final prototype on the basis of literature on GERD and literature on mHealth apps by conducting a thorough literature review and assessing our prototype on the basis of this research.

## 3. Results

### 3.1. Results from the Design Phase

#### 3.1.1. Design Stage 1 Results

**Figure 2:**
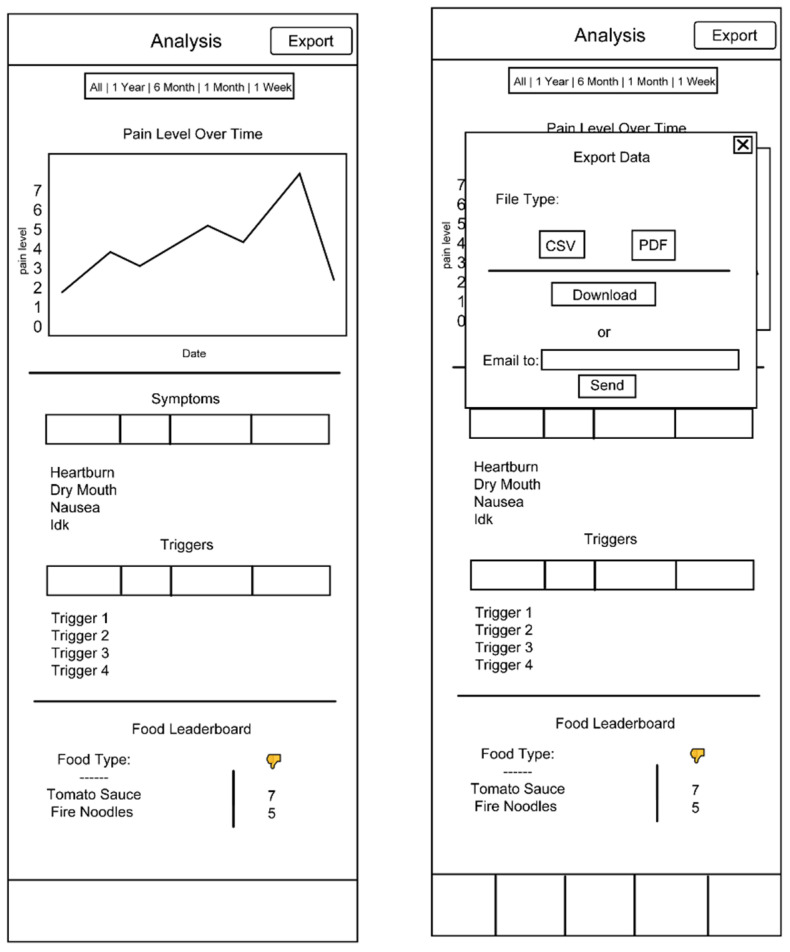
Low-fidelity prototype.

The methods used in Design Stage 1 allowed us to identify four main features that would meet user needs: (1) tracking acid symptoms, (2) analytics and suggestions about a healthier life, (3) exporting the data about symptoms, and (4) finding community information about acid reflux. These features were then incorporated into an initial low-fidelity paper and digital prototype (Figure 2), and each was represented on the bottom navigation bar of the app.

Results from the competitor analysis indicated that exporting symptom data and viewing community information were the most poorly executed features across competitor apps.

To address the first key task of tracking GERD symptoms, our low-fidelity prototype included a salient “add” button (represented as a plus sign) on the app home page, which leads to a page where users can record their triggers and symptoms, including details such as when triggers and symptoms occurred, ingredients/portion (in the case of food triggers), and additional details such as exercise intensity. The layout and design of this tracking feature were inspired by popular and user-friendly health apps, including Bearable and Clue [41,42]. Similarities to existing apps on the market were included to improve efficiency, memorability, and learnability.

A calendar feature was added that would allow users to easily scan symptoms over a long period of time and identify days on which symptoms and triggers have been recorded. Users would be able to click a date on the calendar to view a pop-up list of trigger and symptom recorded on that day, including the types of triggers/symptoms, when these triggers/symptoms occurred, and supplemental data such as food portion size and symptom severity. Clicking a specific day on the calendar would also allow users to edit symptom/trigger data entered for that day.

Our low-fidelity prototype also included an analysis page, which aimed to allow users the flexibility to view simple analytical tools—a symptom-severity-over-time line graph, a top symptoms list, a top triggers list, and a food leader board—for customizable time periods. Importantly, because exporting data was identified as a weakness/pain point for competitor apps, our low-fidelity analysis page included a clearly labeled export button at the top of the screen. When exporting data, users were invited to choose a file type (CSV or PDF) and could either download a report directly on their device or have a report sent to an email address of their choice.

This first version of our prototype also included a community page which directed users to a resource list designed to include expert-vetted web resources where users can access credible information about GERD treatments, GERD research, and access to healthcare. A forum page and option to invite other users to download the app were also included on the community page feature.

Additional design decisions that our team made early in prototyping included choosing color schemes that would make our app stand out from the orange color scheme used in the majority of acid reflux apps available for iOS and Android. Our blue theme was selected to enhance the app’s visibility in app stores. Furthermore, blue was chosen for our app because blue elicits positive, relaxing feelings [43], which was the calming message we wanted to send to our users.

**Figure 3:**
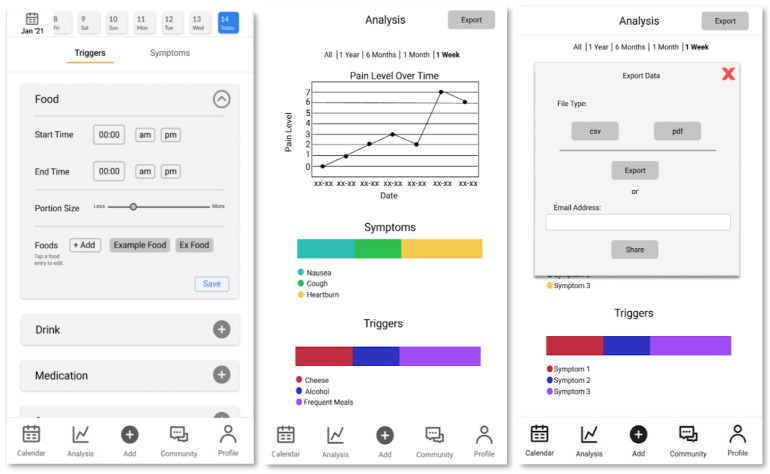
Pages from medium fidelity prototype. From left to right: calendar page, analysis page, export feature, trigger/symptom input page, food trigger ingredient input page, and user profile page.

An important final result of Design Stage 1 was the production, ahead of our first user interviews, of a medium-fidelity prototype on Figma, an online medium-fidelity prototyping tool. This prototype included data visualization, symptom tracking, trigger tracking, and community features (Figure 3).

#### 3.1.2. Design Stage 2 Results

Design Stage 2 focused on incorporating feedback from participants with GERD. Participants commented that they wanted “the ability to log meals” and “[a] note tab because everyone’s different.” Comments like these guided the app development and indicated critical features for the app. 

The consensus seemed to be that the participants wanted to record their acid reflux triggers (such as diet or stressors) with notes after they experienced an episode of acid reflux. Second, they wanted to export their data to share with a healthcare professional. Third, the participants wanted alarms for mealtimes and medications to help regulate their lives. Finally, participants wanted the app to include a community aspect such as a forum or a chat where they could exchange information with other acid reflux patients. Although we had most of these features already in place in our Stage 1 Figma prototype, we added a notes function (Figure S3) to the symptoms tracking feature and added an alarm component (Figure S4) in our app settings. These app features ultimately evolved into an important profile function, which allowed additional app personalization options.

#### 3.1.3. Design Stage 3 Results

Based on the results of the concurrent think-aloud, interview, and survey, we made several changes to the prototype: We added trashcan icons to food trigger ingredients (Figure S5) to make deleting individual ingredients easier for users; we added frequency tables (Figure S6) to the analysis page to show how often the three most frequently reported symptoms were reported in conjunction with frequent triggers; and we added percentages to the symptoms and triggers display for additional context.

Furthermore, users suggested placing a back button in the community section (Figure S7) for easier navigation. In response to feedback on the color scheme of the app, we committed to carrying the same color theme to relevant buttons and headers (Figure S8). We also decided to use circle and square symbols in addition to color differentiation on the calendar pages (Figure S9) to accommodate people with colorblindness.

Four participants responded to the post-interview survey (one participant declined). The post-interview survey asked participants how easy and enjoyable the app was. Survey results indicated that participants found the app moderately easy to use (*n* = 3) and extremely easy to use (*n* = 1). Participants also found the app “neither enjoyable nor unenjoyable” (*n* = 2) and “moderately enjoyable” (*n* = 2).

#### 3.1.4. Design Stage 4 Results

All Stage 4 users had problems with downloading the analysis page; in response, three of them suggested that we remove the export button and move the email function to a secondary pop-up screen. We restructured the download analysis page to have clearer selection methods and allow for multiple actions, such as emailing and downloading, at a time (Figure S10). In addition, two participants had trouble locating the food leaderboard and suggested we move it up and condense the analysis page (Figure S11).

Users also suggested that we implement a dark mode to save power, accommodate diverse environmental conditions, and cater to users who generally prefer working in dark mode. Some users failed to differentiate the forum and expert advice features on the app’s community page, which led us to change the language on the community menu to differentiate between the two features (Figure S12). In response to feedback, we also changed the order of community menu items to place trusted expert resources above the social forum feature (Figure S12). Additionally, we administered a post experiment survey where we asked participants about ease of use and joy of use. Survey results indicated that participants found the app “slightly easy to use” (*n* = 2) and “moderately easy to use” (*n* = 2). Participants also found the app “neither enjoyable nor unenjoyable” (*n* = 2) and “extremely enjoyable” (*n* = 2).

Participants suggested that status messages should confirm that a trigger or symptom had been saved. Some users felt that there were too many steps involved in adding a food trigger; however, we intend to add “Favorites” and “Recents” functions (Figure S5), which were not fully prototyped, to mitigate this problem.

**Figure 4:**
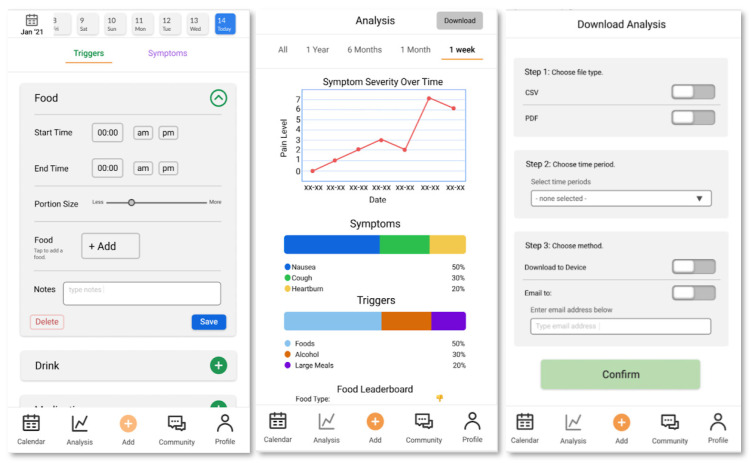
Pages from final medium-fidelity prototype. From left to right: calendar page, analysis page, export feature, trigger/symptom input page, food trigger ingredient input page, and user profile page.

During our final design stage, our team of usability experts employed several methods to address accessibility. First, we used a plugin on Figma called Able, which provides a color contrast ratio and gives a pass/fail score for small text and large text readability. This app received a passing score for text size (Figure S13). We ran additional tests with color blindness simulators and determined that the app was readable. Rather than relying solely on color to convey key information, we utilized color contrast and shape in conjunction with each other to ensure that the app was accessible to colorblind individuals. Furthermore, as mentioned in Section 3.1.2, users have the option in the settings (Figure S14) to change the contrast/theme, background color, and text size to make the app more accessible to colorblind or visually impaired individuals. The final prototype from Design Stage 4 can be seen in Figure 4.

Figures S15–S19 in Supplementary D provide a link to our team’s final Figma prototype, as well as images detailing several of the app’s key sections. 

### 3.2. Results from the Evaluation Phase

#### 3.2.1. Evaluation by Experts in Human-Centered Design

The final app that our team submitted to the 2021 Mobile Health Applications for Consumers Design Competition was the product of direct user feedback and an empirically validated iterative design process. We did not expect iterative improvements to cease after the theoretical launch of our app, and we identified several avenues for future enhancements. Based on user interest, we planned to add a feature to process photos of food and scan barcodes of food triggers. These features would make the app faster to use, help users visualize meals, and provide more accurate information about the food triggers. As an additional time-saving feature, we anticipated adding a widget function to the app, so users could easily access the app and log triggers/symptoms without having to enter the app. We anticipated that user feedback and user testing would guide future iterations of the app and would aim to create a higher fidelity prototype so we could include additional features not available in Figma. Further, we recognize the limitations of the formative user testing methods we employed during the design thinking process described in this paper, and would aim to incorporate summative or quantitative methods, such as A/B testing of key features, surveys with larger sample sizes, and more formal benchmarking (e.g., or error number or task success rates), into research on future iterations of GerdHelper.

Yet, even in light of the fact that our final high-fidelity prototype did not represent a “finished product,” we recognize the potential room for improvement. Judges for the design competition—all doctorate-level experts in human-factors in medicine—provided a number of valuable suggestions related to the design process.

One competition judge pointed out that the somewhat “cartoonish” names chosen for our personas early in the design process may have interfered with our team’s ability to truly empathize with them as part of a user-centered design process. Reviewer comments also addressed the time associated with tracking triggers and symptoms on a day-to-day basis and suggested more innovative solutions to the problem of how GERD patients would fit use of the app into their daily routines.

Reviewers also provided compelling feedback related to the analysis page of our app and its ability to integrate user data over time into a display that is informative and helpful to patients. Because our team did not include any medical professionals, we aimed to create a straightforward analysis of descriptive statistical information and avoided providing inferences or medical advice. This approach leaves users without any specific advice about how to manage the relationship between reported symptoms and triggers or when to seek medical advice. Further, the prototype analysis page does not make clear how multiple symptoms can be tracked over time or how symptom and trigger percentages are calculated. Furthermore, while the analysis page provides a frequency table pairing frequent symptoms with the top three triggers reported on the same day as said symptoms, it is not clear how useful this output would be to GERD patients because a trigger reported at one time of day is not necessarily related to a symptom reported on the same day (perhaps at a different time of day or conceivably even before the trigger occurred). Overall, the analysis would require additional fine tuning ahead of launch.

Another striking suggestion from competition judges was the comment during a post-mortem discussion with one of the judges that our app prototype might be as valuable or more valuable as a research tool for scientists studying the interplay of GERD symptoms and behavior than it would be as a medical tool designed for treating and controlling GERD.

#### 3.2.2. Evaluation on the Basis of GERD-Expert Feedback

Two scientist–practitioners responded to our web-based email survey. The experts were given specific, but open-ended questions to allow them an opportunity to elaborate when needed, but to also obtain specific insights about GERD, our app, and how our app could be used for GERD management. At the end of the survey, they were prompted to give any additional feedback about the app. The experts were given a link to the final prototype in the Qualtrics survey that was emailed to them.

Expert 1 reported being an MD who works as both a practitioner and a research scientist conducting NIH-funding research on both GERD pathogenesis and GERD interventions. Expert 1 agreed with our team’s assessment that the existing GERD apps are inadequate as tools for managing GERD. 

When asked which behavioral interventions are useful for GERD patients, Expert 1 suggested that fitness, leading a healthy lifestyle, adequate sleep time, and controlling anxiety should be top priorities. Expert 1 also suggested that personalized feedback and education materials should be included in an app designed to control GERD. They cited Oura Ring as an example of an app that successfully integrates patient data into personalized feedback.

Expert 1 also provided several useful suggestions about diagnostics. They suggested the GERDQ, a self-assessment diagnostic questionnaire [44], as a useful tool for GERD patients. They also stated that a diary can be a useful exercise for patients who are in the beginning stages of GERD diagnosis and treatment.

After reviewing our prototype, Expert 1 stated that they would potentially recommend our tool to GERD patients, but they also suggested specific improvements, including a mechanism for linking the app to patients’ electronic medical records and a mechanism for incorporating a symptom score. Expert 1 further suggested that medication tracking and exercise should be more prominent features of the app.

Expert 2 reported being an MD who works as a practitioner, a research scientist, and a gastrointestinal expert with 41 years of experience. This expert also reported that they have conducted original and drug company research. They stated that the key behavioral considerations for GERD management are not eating late in the day and elevation of the patient’s heads while they sleep. This expert stated that although food and smoking can trigger GERD symptoms, these two are relatively minor issues in GERD treatment.

After reviewing the GerdHelper app, this expert emphasized the need to educate patients about GERD and GERD risk factors, and they stated that the GerdHelper app may be useful for this purpose. However, they expressed that, while the “younger generation” may find this app useful, they preferred the “old school” style of doctor-to-patient conversation. Furthermore, Expert 2 indicated that the GerdHelper app would be useful for research studies, but they were unsure the degree to which the app would be good for clinical use. Overall, this expert believed that the GerdHelper app might be beneficial to young populations while emphasizing that it is important to keep mHealth tools simple so that the public can actually use them.

#### 3.2.3. Evaluation on the Basis of GERD-Expert Feedback

A rigorous comparison of our final prototype to the literature on GERD and mHealth apps yielded a few important observations about limitations of our prototype. 

First, several issues that currently challenge the mHealth industry as a whole are also apparent in our own design process and prototype. Privacy and security are important challenges in the mHealth literature that were not central to our user-centered design process and are not adequately addressed by GerdHelper’s current features [13]. It would be beneficial to evaluate GerdHelper’s compliance with current medical privacy regulations, as well as conduct further user testing with a focus on how GerdHelper fulfills users’ privacy preferences and expectations.

In addition, as with many other medical apps designed to help patients manage chronic conditions and change behaviors, the app was not the subject of a randomized control trial which should be conducted in the future to provide reliable evidence about its efficacy and usefulness [13,17].

## 4. Discussion

GERD is a prevalent clinical condition that may have serious consequences, including loss of life satisfaction and an increased risk of esophageal cancer [3,4]. We used a variety of empirically supported methodologies and expert insights to design a prototype of the GerdHelper app for the purpose of helping patients to understand and address GERD symptoms. Results from our design and evaluation phases led to valuable insights about what GERD patients want and need in a mobile application.

First, there are no adequate mobile apps to aid patients with GERD, despite the large number of people with GERD. This sentiment is also reflected in the literature [22] and by the experts we interviewed. Second, we identified a set of key features that GERD patients need/want for alleviating and managing their symptoms. These features can be used as a reference point for treatment plans and future app designs for patients with GERD. Third, we documented a process for developing and evaluating a mHealth app following human-centered design guidelines. The procedures and methods we used can also be referenced by future app developers who aim to improve their app design process with human-centered design principles. Furthermore, we have identified resources that app designers can use for accessibility purposes. 

Although we incorporated key features and pain points for GERD patients into our app, we also recognize limitations of the GerdHelper app and areas for improvement. First, due to financial and time limitations, we did not create a high-fidelity prototype with live functions for the participants to use. Instead, the prototype featured a limited number of actions that the participants could perform. However, most features and buttons relevant to our user-testing tasks were clickable and usable. If this app continues into production, a live, more comprehensive prototype would be needed for additional rounds of user testing. Indeed, per feedback from the GERD experts, we believe that more user testing is needed in order to optimize the GerdHelper app. 

We would highly recommend that future rounds of testing include users with a greater range of ages. Our users ranged in age from 18 to 44, which does not reflect the reality that advanced age is associated with an increase in GERD risk, with an emerging patient population between 30 and 39 years old [4]. We also recognize the great importance of testing digital tools on users beyond the researchers’ own social circles in order to minimize personal biases in user feedback. We would also note as a limitation of this study that our expert survey was not formally validated

In line with feedback we received from the Medical Apps for Consumers Design Challenge judges, we would recommend further consideration of time-saving features that might make the app easier to use in addition to continued consideration of more useful ways to present and analyze patient data on the analysis page.

Another important limitation of the GerdHelper app is the lack of input from medical experts throughout the design process. This limitation is illustrated by Expert 1′s suggestion that fitness, sleep, control of anxiety, and a diary should be central aspects of a GERD-management app. In addition, both experts that we surveyed emphasized educational resources as a critical part of GERD education; our prototype did not include highly testable educational sections.

Expert 1 further shared that because each case of GERD is best treated in manner specific to the individual patient’s symptoms and diagnostic history, diagnostic personalization, including the GERDQ [42], would be ideal features of an app for controlling GERD. It would be most beneficial to include medical professionals during a design process, so that appropriate diagnostic tests and medical recommendations can be added.

The fact that mHealth applications which provide medical advice should be, but in practice often are not, designed and vetted by qualified medical experts reflects a troubling reality for the industry. This problem is exemplified by a 2013 study in JAMA Dermatology that scrutinized apps which analyze consumers’ photos of skin lesions and classify them as benign or malignant. Of the mentioned four apps, three provided an inaccurate classification of at least 30% of concerning lesions [13,45]. To complicate matters, the mHealth app industry is currently governed by inadequate standards, certification mechanisms, and government oversight [13]. Appropriate oversight structures would ease the design process for mHealth apps in addition to facilitating higher quality, more useful apps.

Another related issue involves privacy and security concerns surrounding mHealth apps. As discussed above, privacy and security were afterthoughts for our team. We acknowledge, however, the importance of enhancing the privacy and security of mHealth apps by putting these issues at the forefront of the design process—especially in light of inadequate attention to and research on this issue across the mHealth industry [13]. More stringent attention to data privacy and data security would be needed before implementing Expert 1’s recommendations to include diagnostic tools and medical-record-interface options to our app.

Finally, a critical concern about mHealth apps in general and the GerdHelper app in particular is the need for better empirical research on their effectiveness, including randomized control trials and longitudinal studies [13]. To our knowledge, no GERD apps have gone through randomized control trials. Moreover, only few mobile health apps, except in diabetes research, have gone through randomized control trials [12,13,14,15,16]. In contrast with the fast pace of the tech industry where mHealth apps originate, high-quality empirical research on medical interventions requires a long timeline. Reconciling these contrasting approaches to research and development is a challenge that the mHealth industry—including projects such as the GerdHelper app—must meet in order to fulfil their promise to patients and consumers.

## 5. Conclusions

Despite all the app-specific limitations and mobile app limitations, the GerdHelper app provides valuable insights that can be utilized for future app development for chronic conditions such as GERD. The main contribution of this paper is to outline a methodology that mHealth app developers can reference to create a human centered app. This paper will especially provide help to those without a background in user research, in addition to illustrating the challenges that user-research experts face in incorporating medical expertise into the design of mHealth applications.

This article outlines the process, design methods, and evaluation approaches that can be used for future app developers to reference. Human centered design should be the guiding principle for usability and accessibility. A team approach is also recommended for mHealth apps so that end users are well-supported by medical resources, education, personalized feedback, and community support, while utilizing the apps to their maximum capacity. Well-designed apps can drastically improve people’s quality of life and life satisfaction. Our design procedure and research can help others create apps that will impact people’s lives for the better.

## Data Availability

The data used for this study can be found in the following location: https://drive.google.com/drive/folders/1IieJffSiJIUzdcBx397S7e1eM2ynvgTU?usp=sharing (Last accessed: 30 October 2021).

## Supplementary Materials

The following supporting information can be downloaded at: https://www.mdpi.com/article/10.3390/ijerph19031128/s1, Figure S1. Low-fidelity prototype; Figure S2. Low-fidelity prototype; Figure S3. Notes Function; Figure S4. Alarm Component; Figure S5. Trash can icons, favorites, and recently used entries; Figure S6. Frequency Tables; Figure S7. Back button; Figure S8. Color Theme; Figure S9. Calendar Symbols; Figure S10. Analysis Downloa; Figure S11. Food Leaderboard; Figure S12. Community Page Menu; Figure S13. Able Contrast Test; Figure S14. Profile Page; Figure S15. Final Figma Prototype Screens; Figure S16. Final Calendar; Figure S17. Analysis Page; Figure S18. Symptom and Trigger Adding; Figure S19. Community Pages; Table S1. Personas created during Design Stage 1; Table S2. Competitor analysis conducted during Design Stage 1; Table S3. Heuristic evaluation conducted during Design Stage 1; Reference [45] are cited in the Supplementary Materials.

## References

[1] Katzka D., Kahrilas P. (2020). Advances in the Diagnosis and Management of Gastroesophageal Reflux Disease. BMJ.

[2] Sharma P., Yadlapati R. (2021). Pathophysiology and Treatment Options for Gastroesophageal Reflux Disease: Looking beyond Acid. Ann. N. Y. Acad. Sci..

[3] Ness-Jensen E., Hveem K., El-Serag H., Lagergren J. (2016). Lifestyle Intervention in Gastroesophageal Reflux Disease. Clin. Gastroenterol. Hepatol..

[4] Yamasaki T., Hemond C., Eisa M., Ganocy S., Fass R. (2018). The Changing Epidemiology of Gastroesophageal Reflux Disease: Are Patients Getting Younger?. J. Neurogastroenterol. Motil..

[5] Choi J.M., Yang J.I., Kang S.J., Han Y.M., Lee J., Lee C., Chung S.J., Yoon D.H., Park B., Kim Y.S. (2018). Association Between Anxiety and Depression and Gastroesophageal Reflux Disease: Results from a Large Cross-Sectional Study. J. Neurogastroenterol. Motil..

[6] Maret-Ouda J., Markar S.R., Lagergren J. (2020). Gastroesophageal Reflux Disease: A Review. JAMA.

[7] Serena S., Michele R., Chiara M., Gioacchino L., Lorella F., Tiziana M., Gian L.d.A., Francesco D.M. (2018). Relationship between Helicobacter Pylori Infection and GERD. Acta Biomed..

[8] Shibli F., Skeans J., Yamasaki T., Fass R. (2020). Nocturnal Gastroesophageal Reflux Disease (GERD) and Sleep: An Important Relationship That Is Commonly Overlooked. J. Clin. Gastroenterol..

[9] Peery A.F., Dellon E.S., Lund J., Crockett S.D., McGowan C.E., Bulsiewicz W.J., Gangarosa L.M., Thiny M.T., Stizenberg K., Morgan D.R. (2012). Burden of Gastrointestinal Disease in the United States: 2012 Update. Gastroenterology.

[10] Newberry C., Lynch K. (2019). The Role of Diet in the Development and Management of Gastroesophageal Reflux Disease: Why We Feel the Burn. J. Thorac. Dis..

[11] Tosetti C., Savarino E., Benedetto E., De Bastiani R. (2021). Study Group for the Evaluation of GERD Triggering Foods Elimination of Dietary Triggers Is Successful in Treating Symptoms of Gastroesophageal Reflux Disease. Dig. Dis. Sci..

[12] Anzaldo-Campos M.C., Contreras S., Vargas-Ojeda A., Menchaca-Díaz R., Fortmann A., Philis-Tsimikas A. (2016). Dulce Wireless Tijuana: A Randomized Control Trial Evaluating the Impact of Project Dulce and Short-Term Mobile Technology on Glycemic Control in a Family Medicine Clinic in Northern Mexico. Diabetes Technol..

[13] Kao C.-K., Liebovitz D.M. (2017). Consumer Mobile Health Apps: Current State, Barriers, and Future Directions. PM R.

[14] Lee J.-A., Choi M., Lee S.A., Jiang N. (2018). Effective Behavioral Intervention Strategies Using Mobile Health Applications for Chronic Disease Management: A Systematic Review. BMC Med. Inform. Decis. Mak..

[15] Liu K., Xie Z., Or C.K. (2020). Effectiveness of Mobile App-Assisted Self-Care Interventions for Improving Patient Outcomes in Type 2 Diabetes and/or Hypertension: Systematic Review and Meta-Analysis of Randomized Controlled Trials. JMIR Mhealth Uhealth.

[16] De Ridder M., Kim J., Jing Y., Khadra M., Nanan R. (2017). A Systematic Review on Incentive-Driven Mobile Health Technology: As Used in Diabetes Management. J. Telemed. Telecare.

[17] Zolfaghari M., Shirmohammadi M., Shahhosseini H., Mokhtaran M., Mohebbi S.Z. (2021). Development and Evaluation of a Gamified Smart Phone Mobile Health Application for Oral Health Promotion in Early Childhood: A Randomized Controlled Trial. BMC Oral Health.

[18] Covolo L., Ceretti E., Moneda M., Castaldi S., Gelatti U. (2017). Does Evidence Support the Use of Mobile Phone Apps as a Driver for Promoting Healthy Lifestyles from a Public Health Perspective? A Systematic Review of Randomized Control Trials. Patient Educ. Couns..

[19] Agarwal P., Gordon D., Griffith J., Kithulegoda N., Witteman H.O., Sacha Bhatia R., Kushniruk A.W., Borycki E.M., Lamothe L., Springall E. (2021). Assessing the Quality of Mobile Applications in Chronic Disease Management: A Scoping Review. NPJ Digit. Med..

[20] Chib A., van Velthoven M.H., Car J. (2015). MHealth Adoption in Low-Resource Environments: A Review of the Use of Mobile Healthcare in Developing Countries. J. Health Commun..

[21] Design Competition. https://www.hcs2021.org/app-competition.

[22] Bobian M., Kandinov A., El-Kashlan N., Svider P.F., Folbe A.J., Mayerhoff R., Eloy J.A., Raza S.N. (2017). Mobile Applications and Patient Education: Are Currently Available GERD Mobile Apps Sufficient?. Laryngoscope.

[23] Dam R.F., Siang T.Y. 5 Stages in the Design Thinking Process. https://www.interaction-design.org/literature/article/5-stages-in-the-design-thinking-process.

[24] Owen C. (2007). Design Thinking: Notes on Its Nature and Use. Des. Res. Q..

[25] Cooper A., Arend U., Eberleh E., Pitschke K. (1999). The Inmates Are Running the Asylum. Software-Ergonomie ’99: Design von Informationswelten.

[26] Guo L., Sharma R., Yin L., Lu R., Rong K. (2017). Automated Competitor Analysis Using Big Data Analytics: Evidence from the Fitness Mobile App Business. Bus. Process. Manag. J..

[27] Donaldson D. (2021). Acid Reflux Diet Helper. https://play.google.com/store/apps/details?id=com.appstronautstudios.acidrefluxhelper.

[28] SkyGazer Labs (2021). mySymptoms Food Diary & Symptom Tracker. https://play.google.com/store/apps/details?id=com.sglabs.mysymptoms.

[29] Softarch Technologies AS (2021). Refluxlog—Acid Reflux and Heartburn Trigger Log. https://play.google.com/store/apps/details?id=com.RefluxLog.app.

[30] Nielsen J. Enhancing the Explanatory Power of Usability Heuristics. Proceedings of the SIGCHI Conference on Human Factors in Computing Systems.

[31] Nielsen J. Finding Usability Problems through Heuristic Evaluation. Proceedings of the SIGCHI Conference on Human Factors in Computing Systems.

[32] Weinschenk S., Barker D. (2000). Designing Effective Speech Interfaces.

[33] Adams W. (2015). Conducting Semi-Structured Interviews. Handbook of Practical Program Evaluation.

[34] Blandford A. Semi-Structured Qualitative Studies. https://www.interaction-design.org/literature/book/the-encyclopedia-of-human-computer-interaction-2nd-ed/semi-structured-qualitative-studies.

[35] Gemayel T. How to Wireframe. Figma. 12 August 2019. https://www.figma.com/blog/how-to-wireframe/.

[36] Nielsen J., Landauer T.K. A Mathematical Model of the Finding of Usability Problems. Proceedings of the ACM INTERCHI’93 Conference.

[37] Fonteyn M.E., Kuipers B., Grobe S.J. (1993). A Description of Think Aloud Method and Protocol Analysis. Qual Health Res..

[38] Alshammari T., Alhadreti O., Mayhew P.J. (2015). When to Ask Participants to Think Aloud: A Comparative Study of Concurrent and Retrospective Think-Aloud Methods. Int. J. Hum. Comput. Interact..

[39] Michaelidou N., Dibb S. (2006). Using Email Questionnaires for Research: Good Practice in Tackling Non-Response. J. Target. Meas Anal. Mark..

[40] Scheuren F. (2004). What Is a Survey.

[41] Bearable (2021). Bearable—Symptoms & Mood Tracker. https://play.google.com/store/apps/details?id=com.bearable.

[42] BioWink GmbH (2021). Clue Period & Cycle Tracker. https://play.google.com/store/apps/details?id=com.clue.android.

[43] Elliot A.J., Maier M.A. (2014). Color Psychology: Effects of Perceiving Color on Psychological Functioning in Humans. Annu. Rev. Psychol..

[44] Suzuki H., Matsuzaki J., Okada S., Hirata K., Fukuhara S., Hibi T. (2013). Validation of the GerdQ Questionnaire for the Management of Gastro-Oesophageal Reflux Disease in Japan. United Eur. Gastroenterol. J..

[45] Wolf J.A., Moreau J.F., Akilov O., Patton T., English J.C., Ho J., Ferris L.K. (2013). Diagnostic Inaccuracy of Smartphone Applications for Melanoma Detection. JAMA Derm..

