# Supplementary material for: Reviewing the User-Centered Design Process for a Comprehensive Gastroesophageal Reflux Disease (GERD) App
Source: Int J Environ Res Public Health. 2022 Jan 20;19(3):1128. doi: 10.3390/ijerph19031128 (PMC8834221; doi:10.3390/ijerph19031128)
Supplement: Supplementary file 1 [file ijerph-19-01128-s001.zip › ijerph-1465317-supplementary.pdf]

International Journal of
Environmental Research and Public Health

MDPI

Supplementary A

During Design Stage 1, we first created personas, then conducted a competitor analysis and heuristic evaluation of existing GERD and food symptom tracking mobile applications. Our findings from these, along with guidance from our personas, were used when we sketched a low-fidelity prototype (Figure S1). We chose to develop personas because research demonstrates that they are useful for conveying user needs throughout the process of designing medical tools [45]. The competitor analysis was used to see how the chosen features of each app performed compared to the other apps. We then further investigated the usability of those chosen features using a heuristic evaluation.

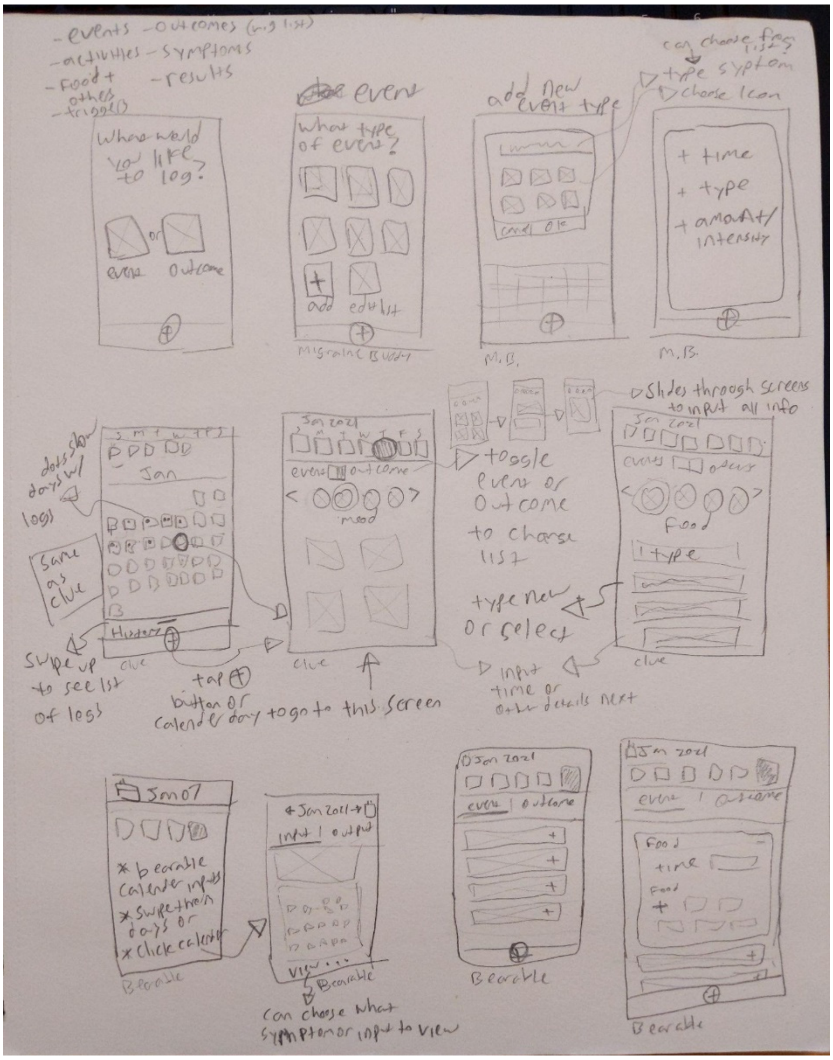

Figure S1. Low-fidelity prototype.

Table S1. Personas created during Design Stage 1.

Persona 1		
**Smoker Samuel**	Description	Smoker Sam is an overweight 48-year-old man who has been smoking for the past 30 years. His wife used to help him manage his acid reflux, but she just started a time-consuming new job and can’t help anymore. He regularly sees a doctor who asks him about his symptoms.
	Needs	He needs to be able to enjoy everyday activities by minimizing or eliminating his GERD symptoms.

	Motivators	Reduce everyday pain and decrease discomfort by controlling his diet, alcohol consumption, and tobacco use.
	Goals	Find tools that track GERD symptoms and reduce the frequency and intensity of GERD flare ups. Share data with his doctor and receive suggestions to avoid and reduce GERD symptoms.
	**Persona 2**	
**Pregnant Patricia**	Description	Patricia is a 32-year-old accountant expecting her first child. Lately, she and her partner struggle to find time to eat right and exercise. Patricia has been experiencing painful heartburn at night and is desperate for relief.
	Needs	She needs to control her acid reflux in order to feel comfortable and stay well-rested throughout the rest of her pregnancy.
	Motivators	Aside from reducing her level of discomfort, she is motivated to attain good health for both herself and her baby.
	Goals	Reduce the pain and discomfort associated with acid reflux and increase the duration and quality of her sleep
	**Persona 3**	
**Stressed-Out Sophia**	Description	Sophia is a 25-year-old woman who works a full-time, high-stress job and is working on an MA degree at the same time. She worries that she might have acid reflux due to her unhealthy diet, but she isn't sure.
	Needs	She needs confirmation that her acid reflux is real and wants something easy and fast.
	Motivators	She wants to get more information about her symptoms so she can manage her diet and minimize health impacts.
	Goals	Track pain and get information about acid reflux and how to manage it.

Once we identified the needs, motivators, and goals of our potential users, we identified four main features that would meet user needs: (1) tracking acid symptoms, (2) analytics and suggestions about a healthier life, (3) exporting the data about symptoms, and (4) finding community information about acid reflux. Our team performed a competitor analysis to evaluate each key feature on three existing apps: (1) Acid Reflux Diet Helper; (2) mySymptoms Food Diary & Symptom Tracker; and (3) Reflux Log. There were three possible ratings for the competitor analysis. Good indicated that the app was successful in the task. Okay indicated that the app was somewhat successful in the task. Poor indicated that the app was not successful in the task. Overall, we found that none of the apps were successful in all four tasks, and there was much room for improvement.

Table S2. Competitor analysis conducted during Design Stage 1.

App	Task 1	Task 2	Task 3	Task 3
Acid Reflux Diet Helper	Good	Good	Poor	Okay
mySymptoms Food Diary & Symptoms Tracker	Good	Okay	Good	Poor
Reflux Log	Okay	Poor	Poor	Poor

After completing the competitor analysis, our four team members, as usability experts, independently completed heuristic evaluations in accordance with research showing that heuristic evaluation by three to five usability experts is an effective method of detecting usability problems [31]. Our team scored on a scale of 1 (completely unsatisfactory) to 10 (completely satisfactory) on each app's capacity to perform four specific tasks. During this process, we identified usability issues and potential solutions. We evaluated the apps and tasks based on six heuristics: learnability, efficiency, memorability, satisfaction, effectiveness, and forgiveness [31,30,32].

Our team evaluated the following tasks: Task 1: track GERD symptoms; Task 2: provide suggestions about lifestyle; Task 3: export symptom data; and Task 4: view community information. As shown in Table S3, we generally assigned low scores across apps and across tasks. We identified Task 3 and Task 4—export symptom data and view community information, respectively—as the most poorly executed features across current apps.

Table S3. Heuristic evaluation conducted during Design Stage 1.

App	Task 1	Task 2	Task 3	Task 4	Average
Acid Reflux Diet Helper	7.83	7.88	1.08	6.04	5.71
mySymptoms Food Diary & Symptoms Tracker	7.84	5.59	7.21	0	5.16
Reflux Log	4.96	0	0	0	1.24
Average	6.88	4.49	2.76	2.02	

Based on our personas, competitor analysis, and heuristic analysis, we created a medium-fidelity prototype on Figma, an online medium-fidelity prototyping tool, with data visualization, symptom tracking, trigger tracking, and community features (Figure S2). We drew inspiration for our prototype from popular and user-friendly health apps, including Bearable and Clue.

Bearable is a health tracking app which allows users to input a large variety of factors to track over time, such as mood, symptoms, sleep, medications, and foods. Similarly, to the app we aimed to design, these factors all require different types of information to be input, making it difficult to use a consistent design from factor to factor. Bearable navigated this problem by listing all factors on a single scrollable page and utilizing drop down boxes to input information for each factor. Tapping on a factor opens a drop-down box where users can input all relevant information for that factor and tapping on the factor again closes the drop-down box, allowing users to easily navigate between factors and accommodating the different information needed for inputs.

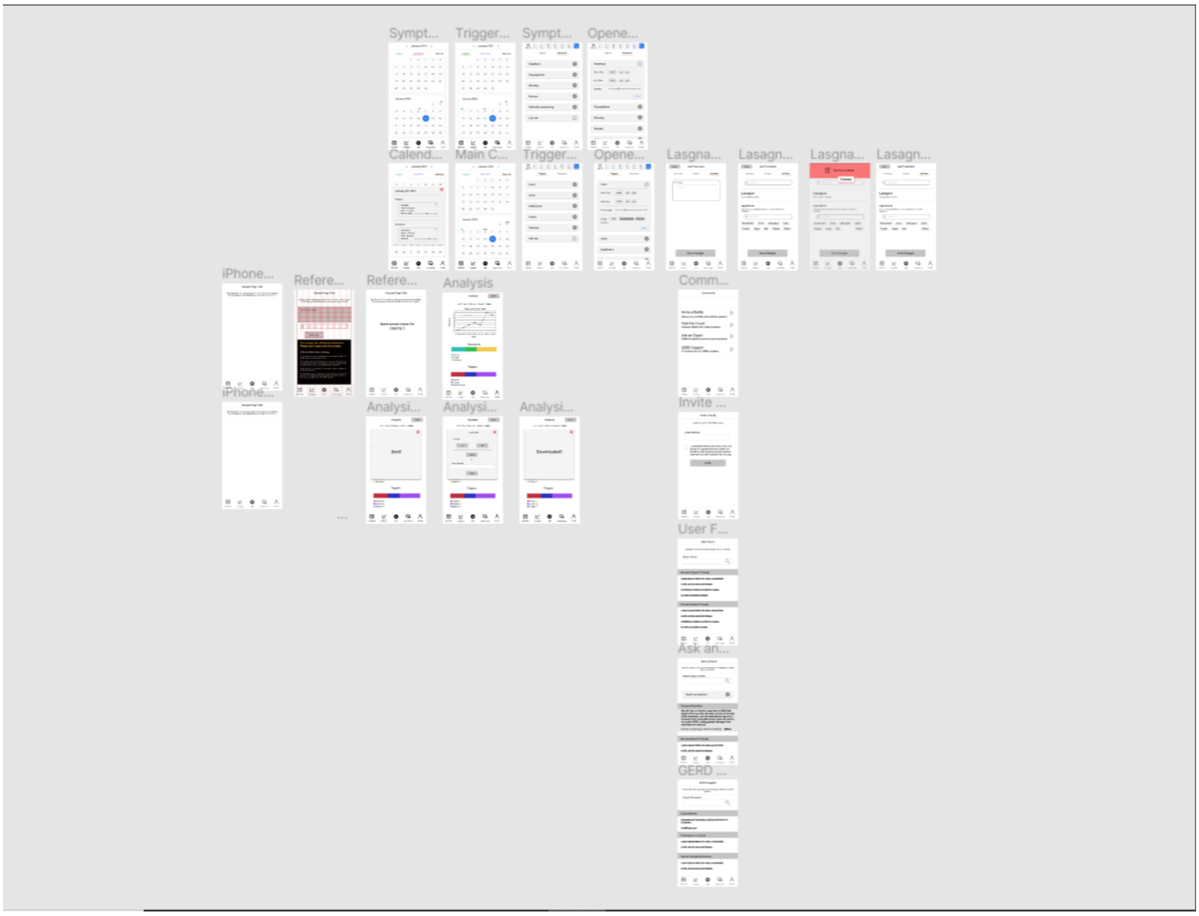

Figure S2. Low-fidelity prototype.

Supplementary B

During Design Stage 2, we interviewed individuals with GERD to understand their needs and determine what they might be looking for in a GERD app. Five participants participated in a semi-structured interview. Ahead of the interview, we asked them to provide answers to the following demographic questions:

1. How old are you?
2. What is your gender?
3. What's the highest level of education you've completed?
4. What is your profession?
5. What is your income level?
6. Have you ever experienced acid reflux symptoms?
7. For how long have you experienced acid reflux symptoms?
8. How often do you experience acid reflux symptoms?
9. How much do your acid reflux symptoms bother you?
10. Do you take medications for your acid reflux?
11. How comfortable are you with navigating smartphone apps?
12. What do you usually use your smartphone for?
13. What kind of applications do you usually use?

Then, participants were asked the following questions as part of a semi-structured interview. Due to the semi-structured nature of the interview, the research team took the opportunity to probe interesting points that came up organically during their conversation with participants and was not entirely limited to these questions:

1. Have you ever used any applications to help manage your health (or body) before?

2. (If they have used such apps) How often do you use these apps?
3. (If they have used) How helpful do you find these apps?
4. How do you control your acid reflux?
5. What behaviors do you think are important for controlling acid reflux?
6. What information do you think is important for controlling acid reflux?
7. Do you currently track anything to help you control your acid reflux?
8. If yes, how do you track it? (I.e., What medium do they use?)
9. Would you use an app to control acid reflux?
10. What features do you think are important for an app used to control acid reflux?
11. Would an app having ads impact whether or not you would use the app? (Or some other question about using apps with ads)
12. Would you pay for an ad-free acid reflux app? How much?
13. How often do you pay for an app? (For access or subscription?)

Specific improvements to the GerdHelper prototype that were made in response to the data gathered during Design Stage 2 included adding a notes function to the prototype, as shown in Figure S3.

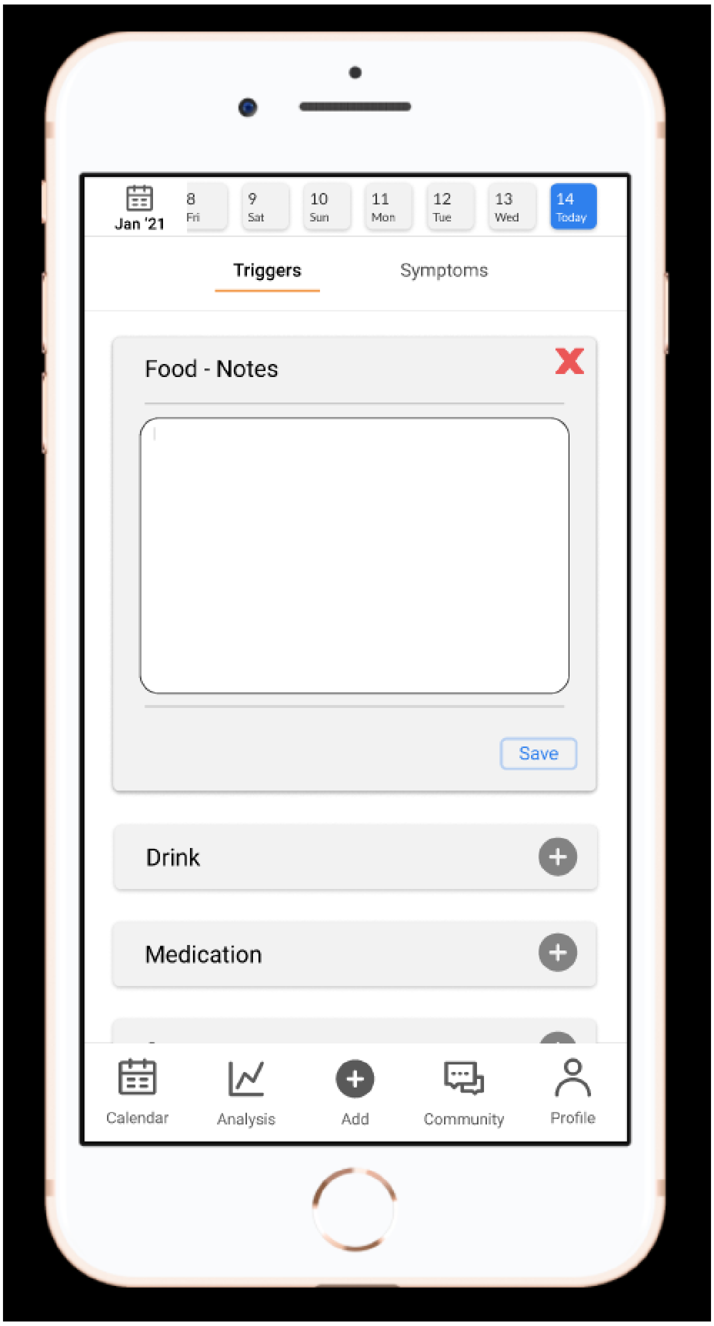

Figure S3. Notes Function.

As shown in figure S4, we also added an alarm component to our prototype based on information gathered during Design Phase 2 user interviews.

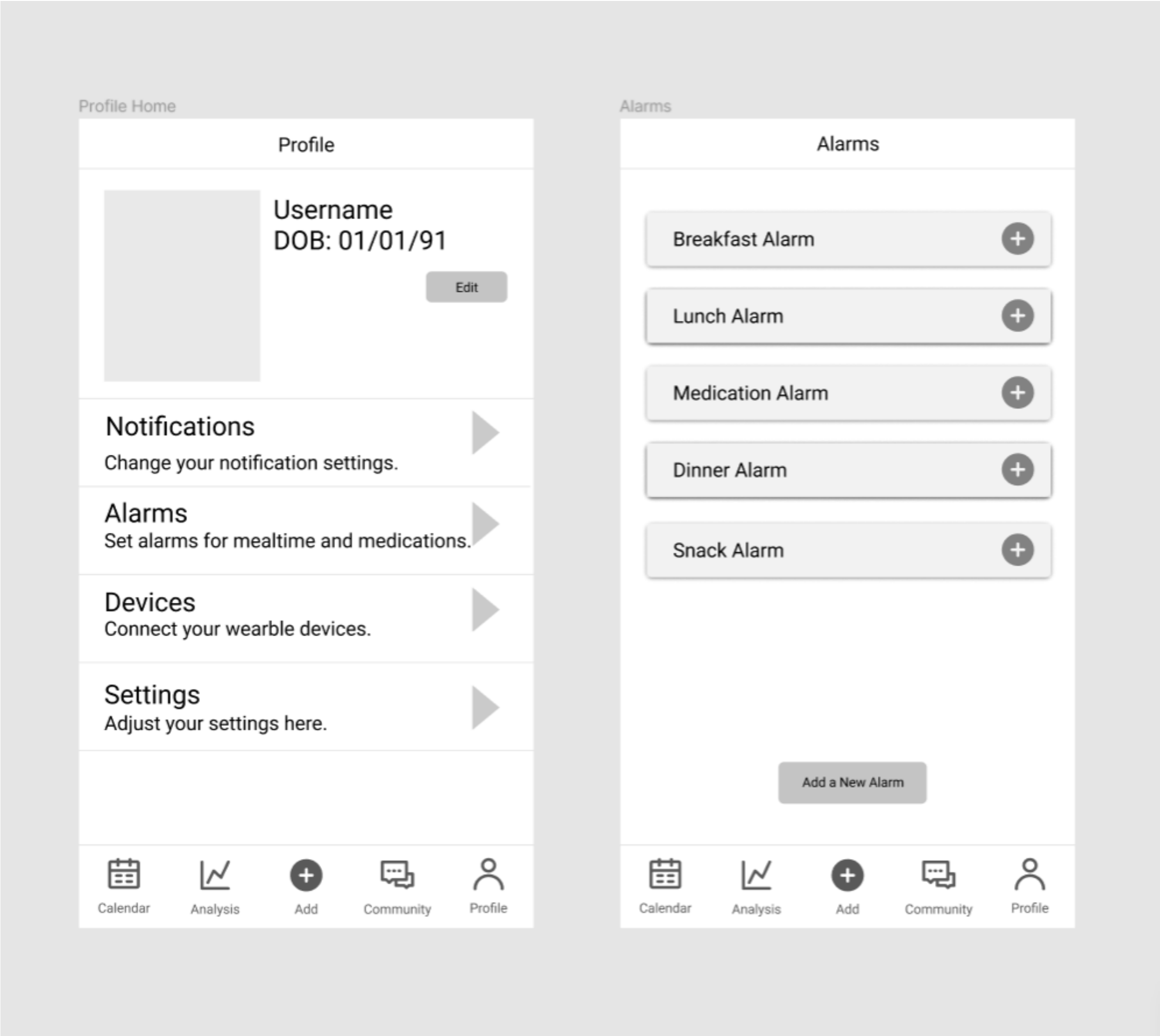

Figure S4. Alarm Component.

Supplementary C

During Design Stage 3, we observed users interacting with our prototype and refined the prototype according to their performance and feedback. Five users were first asked to complete a pre-interview survey that included informed consent and demographic questions:

1. How old are you?
2. What is your gender?
3. What is the highest level of education you've completed?
4. What is your profession?
5. What is your income level?
6. Have you ever experienced acid reflux symptoms?
7. For how long have you experienced acid reflux symptoms?
8. How often do you experience acid reflux symptoms?
9. How much do your acid reflux symptoms bother you?
10. Do you take medications for your acid reflux?
11. How comfortable are you with navigating smartphone apps?
12. What do you usually use your smartphone for?

13. What kind of applications do you usually use?

Next, each participant completed a concurrent think-aloud while using the GerdHelper prototype, so that our team could see how they interacted with the app, where they had trouble using the app, and what they might have been thinking as they used the app, especially at points where they struggled to understand the interface. The following instructions were provided for the concurrent think-aloud:

> *I am going to share with you a prototype for an app designed to help patients track and control acid reflux symptoms. I will ask you to complete several tasks using the prototype. In order to get a feel for how you are using the app, I'd like you to narrate your thoughts about the interface as you are using it. It doesn't have to be too formal—just tell me what you are doing as you are doing it. Tell me what you expect to happen and whether what happens matches your expectations. For example, if I were asked to do this task for making a phone call on my cell phone, I might say something like, "Hm … I've never used a phone like this. I see a phone icon. I think that must be what I am supposed to click on in order to make a call. Yup, now I see a number pad, so I guess I'll enter the number I want to call … (etc)."*

After giving participants these instructions, we asked them to complete the following tasks using GerdHelper:

1. Please use the app to add the symptom "heartburn" for today.
2. Please use the app to add the trigger "lasagna" for today.
3. Please use the app to delete the trigger "cheese" today.
4. Navigate to the calendar view. On which days did the user enter triggers? On which days did they enter symptoms?
5. Please tell me which triggers and symptoms were input for January 6.
6. Use the the analysis feature to identify a food that the user should avoid.
7. Use the analysis feature to identify the symptom that is recorded to be experienced the most often.
8. Download a copy of the analysis page.
9. Visit the user forum and find an entry about GERD during pregnancy.

We asked participants to complete a second navigation task without a think-aloud component, so that we could observe how users might interact with the GerdHelper app without the distraction of narrating their activities. Before the navigation task, we gave participants the following instructions:

> *I am going to ask you to complete several tasks using the prototype. You may have already completed some of them before. This time, I don't want you to talk to me about what you're doing. Please just complete the task as you would if you were working alone.*

Next, we asked participants to complete the following tasks using GerdHelper:

1. Please use the app to add the symptom "heartburn" for today.
2. Please use the app to add the trigger "lasagna" for today.
3. Navigate to the calendar view. Is there a recorded symptom on January 3?
4. Is lemonade on the list of foods you should avoid?
5. Which symptom is the second most experienced symptom?
6. Download a copy of the analysis page.
7. Visit the user forum and find an entry about GERD and pregnancy.

After participants had completed the seven tasks above, we interviewed them about ther perceptions of GerdHelper and their opinions abut the app. We asked participants the following questions:

1. Could you summarize the purpose of this app in your own words?
2. Did you find any of the navigation confusing?
3. Was there anything (else) about the app you found confusing?
4. How similar or dissimilar was the navigation in this app compared to apps you have used in the past?
5. Of the tasks you just completed, were any particularly intuitive to do? Why?
6. Of the tasks you just completed, were any particularly unintuitive to do? Why?
7. I noticed you did ___, can you tell me why?
1. Did you notice whether there was any other way to ____ (email a report, access the calendar)?
2. Can you tell me what you think of the aesthetics of the app?
3. May ask about icon choices, locations of buttons, text choice and size, colors, any navigation animations, analysis displays (chart, lists, bar graphs), aesthetics, etc.
4. What did you think of the layout of the content?
5. Which features of this app would you use the most?
6. Which features of this app would you use the least?
7. Are there any features you think we should add?
8. Was it easy to understand how you would view your symptoms and triggers from past days?
9. Is there anything missing on the triggers and symptoms page?
10. What are your thoughts about the analysis page?
11. What are your thoughts about the community page?
12. If you could change anything about the app, what would it be? Why?

Finally, we sought more insight into participants' impressions of the app via a post-test survey, consisting of the following 10 questions:

1. How easy was this app to use? (7 pt scale)
2. How enjoyable did you find this app to use? (7 pt scale)
3. How likely would you be to use this app on your own? (7 pt scale)
4. How often would you use this app? (7 pt scale)
5. How likely would you be to pay for this app? (7 pt scale)
6. If they would pay for it, How much would you be willing to pay for this app? (freeform)
7. Would the app having ads impact whether or not you would use it?
8. What are some factors that would make it ok for there to be ads?
9. How likely is it that you would recommend this app to a friend/colleague/family member with acid reflux?
10. What would you rate this app overall? (1-10)

Participants' responses to the survey and interview questions above, as well as their observed performance on user testing tasks, led us to make several revisions and additions to the GerdHelper protogype.

Because users struggled to determine how to delete specific trigger-food ingredients in order to customize food trigger entries, we added trash can icons to make the task more intuitive, as shown in Figure S5.

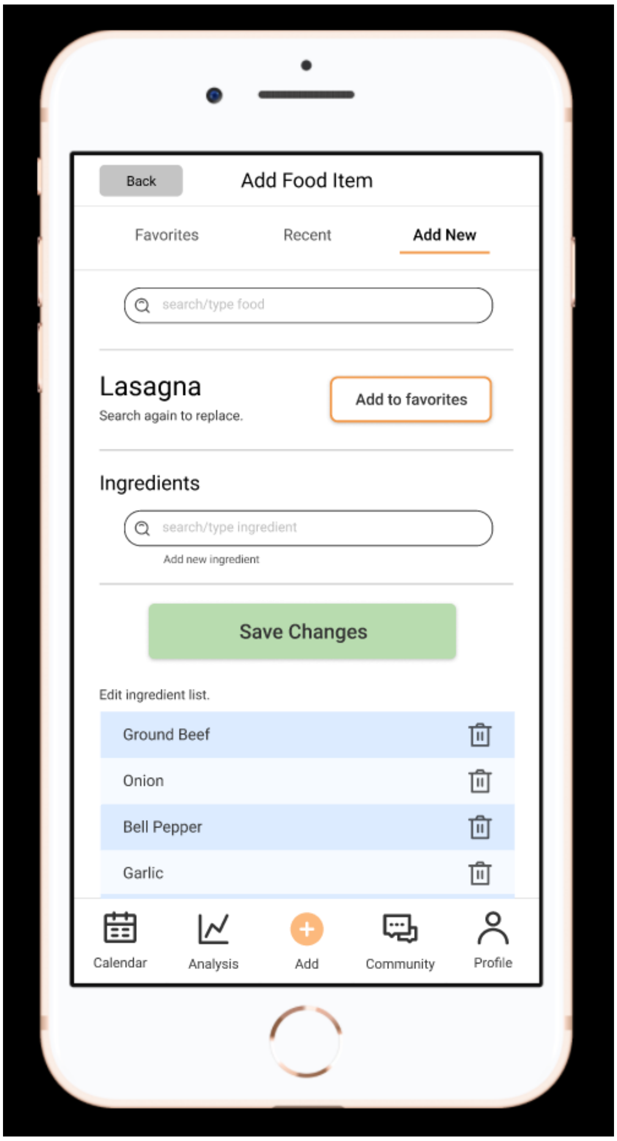

Figure S5. Trash can icons, favorites, and recently used entries.

Users we spoke to during Design Stage 3 expressed frustration in their efforts to interpret data shown on GerdHelper's analysis page. In an effort to help users understand and make use of the analysis page, we added frequency tables that show how often each user's three most frequently reported symptoms were reported in conjunction with frequent triggers, as shown below in Figure S6.

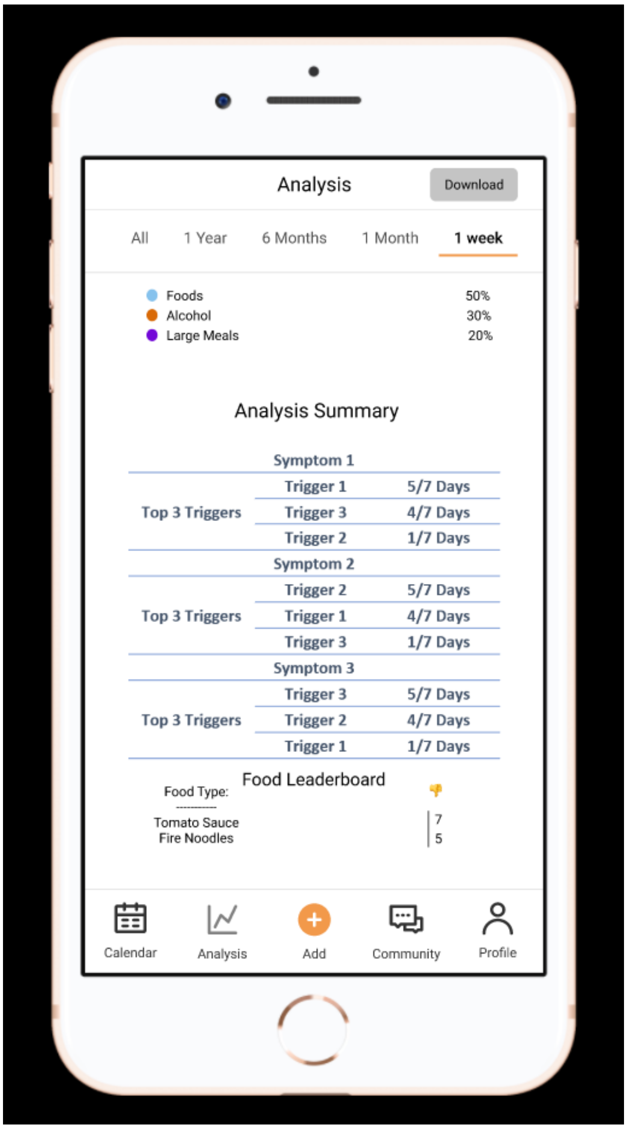

Figure S6. Frequency Tables.

During Stage 3, users suggested that a back button should be placed in the community section of the app. Figure S7 shows the back button.

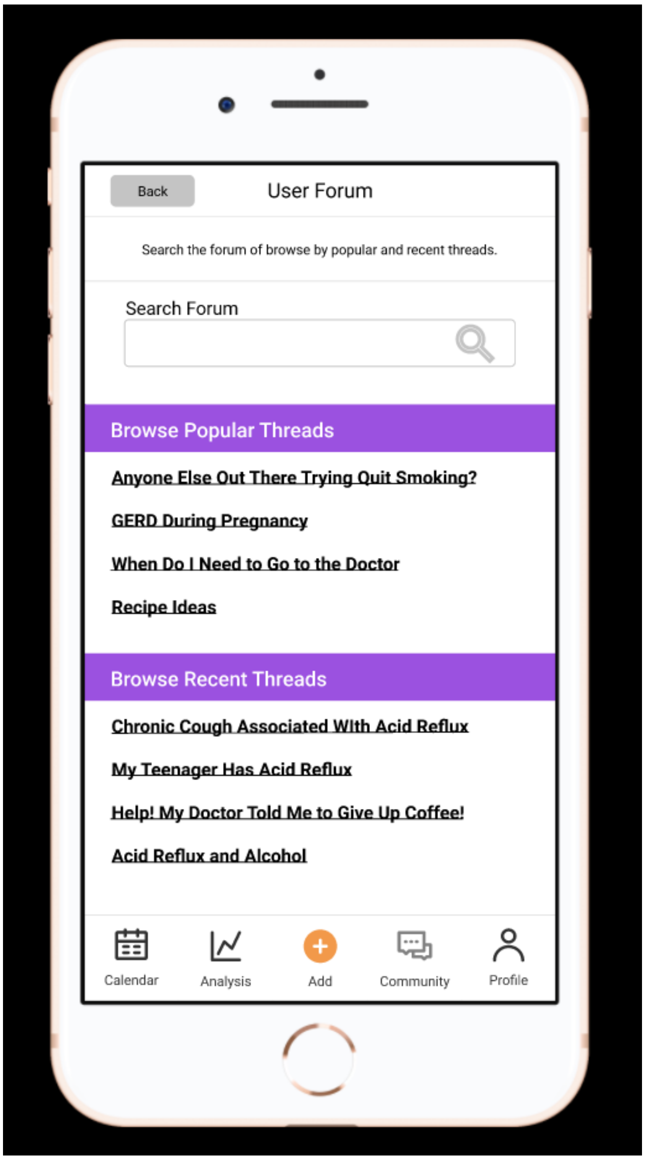

Figure S7. Back button.

Users pointed out inconsistencies in GerdHelper's color scheme across different sections of the app. Therefore, during Design Stage 3 we committed to carrying the same color themes, button formats, and header formats throughout the app in order to create a more professional and aesthetically pleasing experience. Figure S8 illustrates this color scheme.

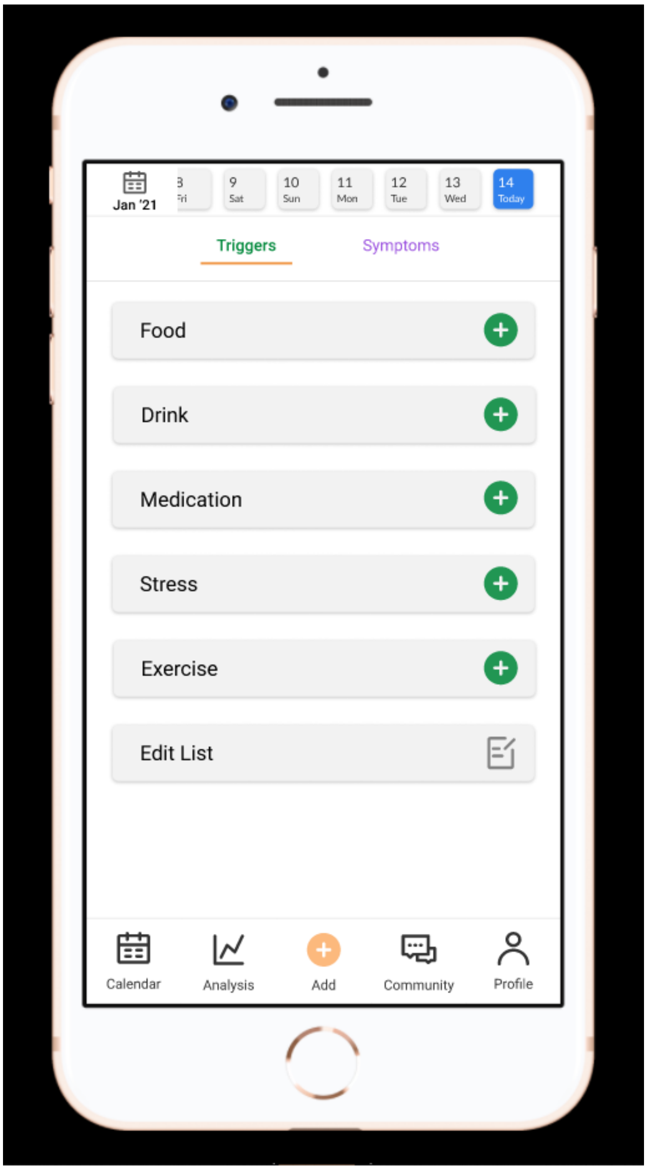

Figure S8. Color Theme.

During Design Stage 3, we revised GerdHelper's calendar page in order to make it easlier for people with color blindness to navigate. Rather than differentiating symbols denoting the entry of triggers and symptoms only with color codes, we took the further step of representing triggers as circles and symptoms as squares in order. Figure S9 illustrates this revision.

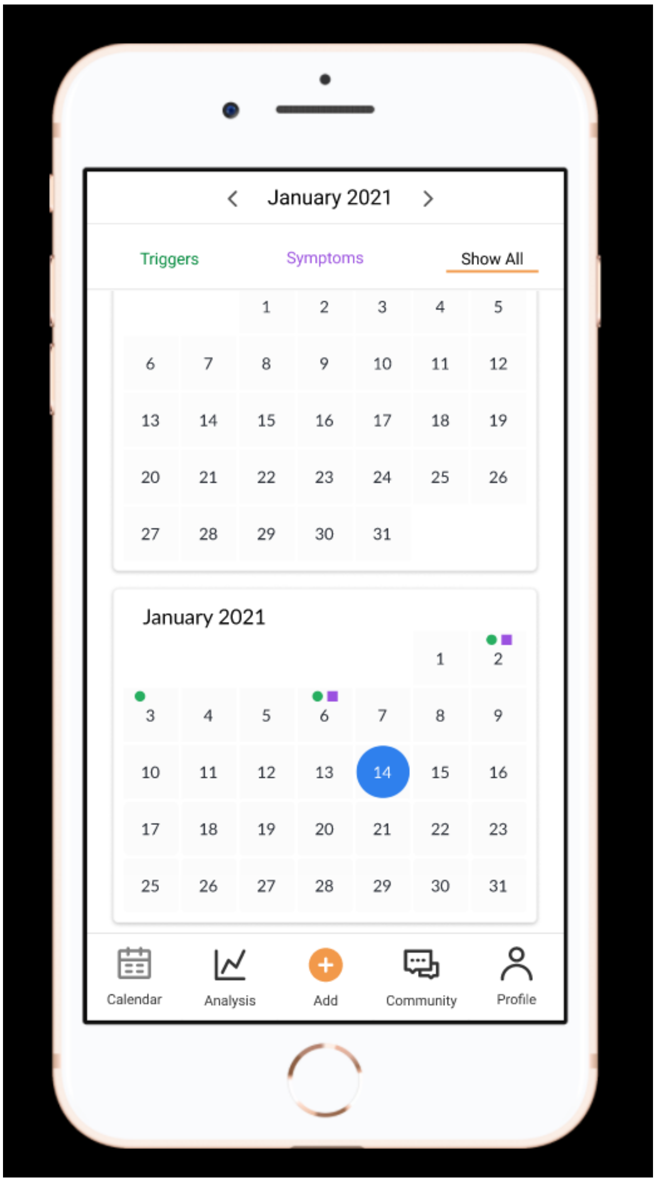

Figure S9. Calendar Symbols.

Supplementary D

During Design Stage 4, we asked a new group of participants to complete the same tasks assigned to users in Design Stage 3. The surveys, interviews, and user testing activities informed additional changes to the GerdHelper prototype during Design Stage 4. One of these changes involved GerdHelper's data download and data sharing functions. Participants struggled to download and email data from GerdHelper during Design Stage 4, so we took the step of restructuring the download page so as to explicitly instruct users about the steps involved in downloading and sharing data, as shown in Figure S10. In addition, we added a "Confirm" button at the end of this process because Design Stage 4 users told us that they were not able to determine when they were finished with the data download task.

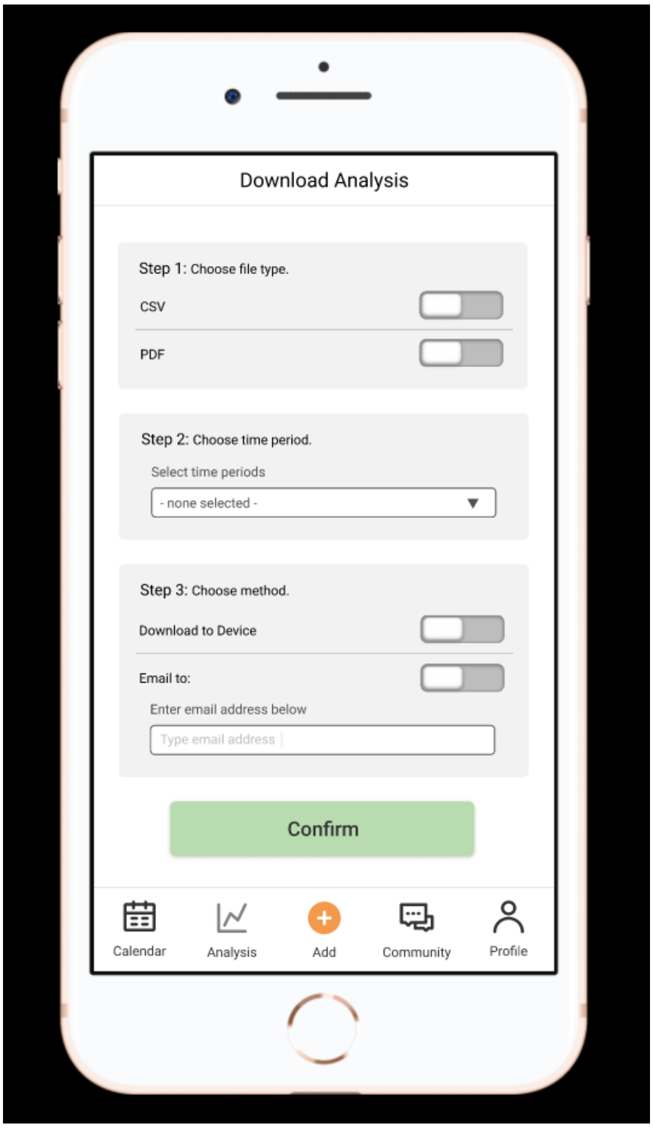

Figure S10. Analysis Download.

Two Design Stage 4 participants had trouble locating the food leaderboard and requested that we move it up and condense the analysis page to make information easier to located. We accomodated these requests, as illustrated in Figure S11.

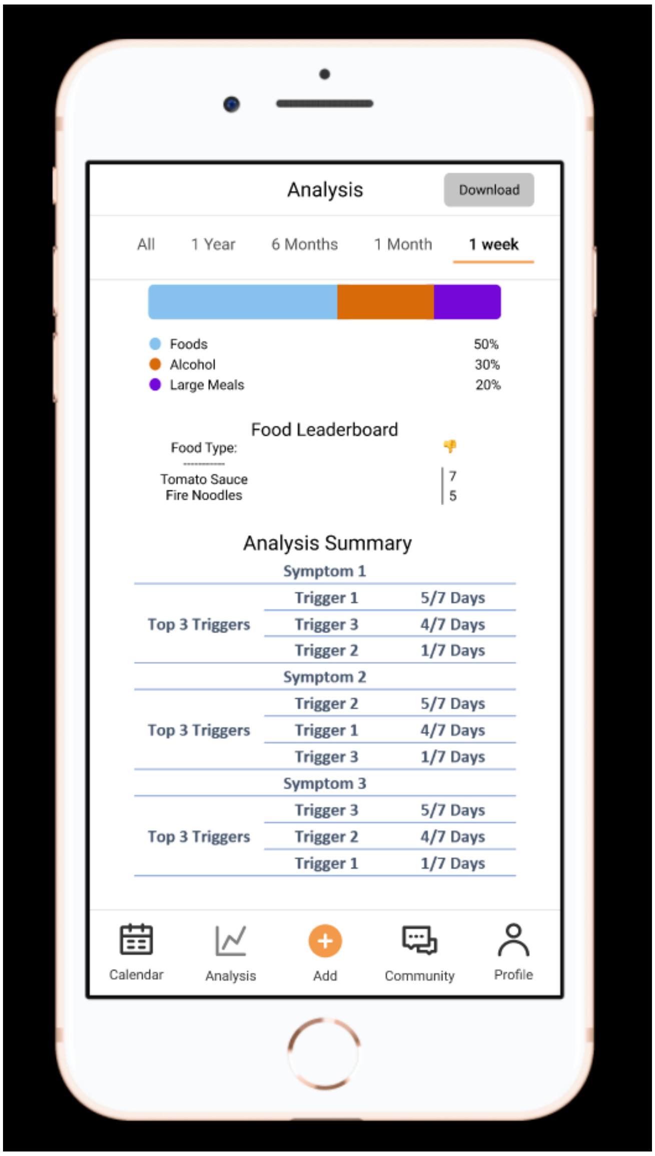

Figure S11. Food Leaderboard.

During Design Stage 4, we made two changes to the community page in response to user feedback: First, we changed the langauge on the menu to help users distinguish between the GERD forum, which allows patients to communicate with each other, and expert advice resources. Second, we moved the expert-vetted GERD Resources feature above the form for the purpose of putting trustworthy, vetted information front and center.

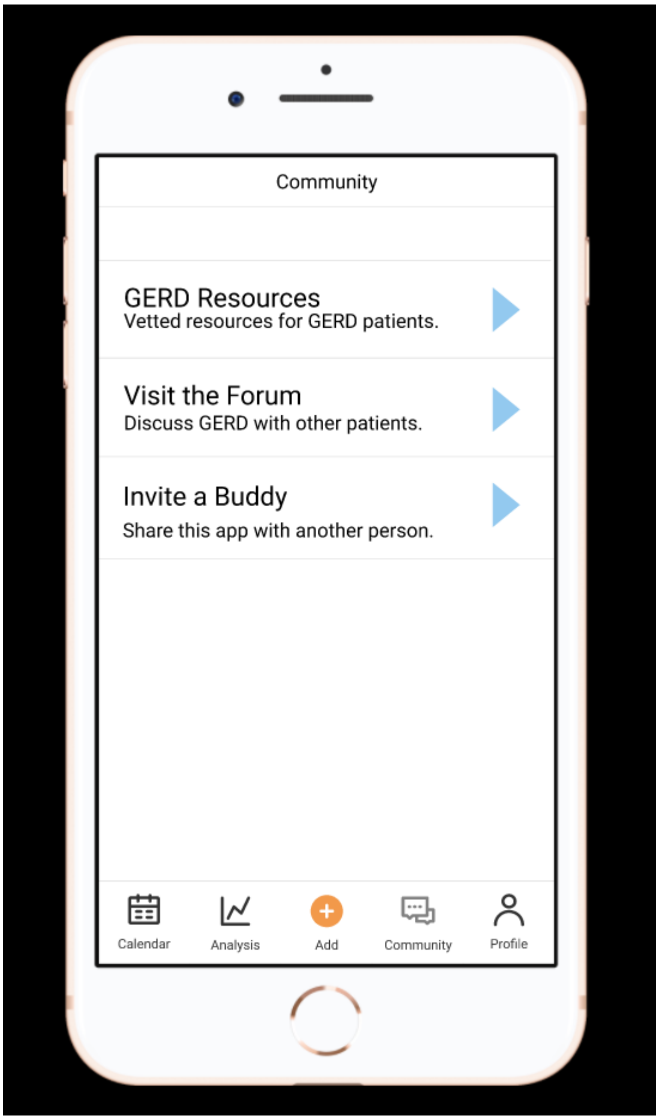

Figure S12. Community Page Menu.

As shown in Figure S13, GerdHelper received a passing score from the Figma plugin Able. Able guides designers towards more inclusive products by analyzing color contrast with the color-blind population in mind, in addition to evaluating the readability of text size within prototypes.

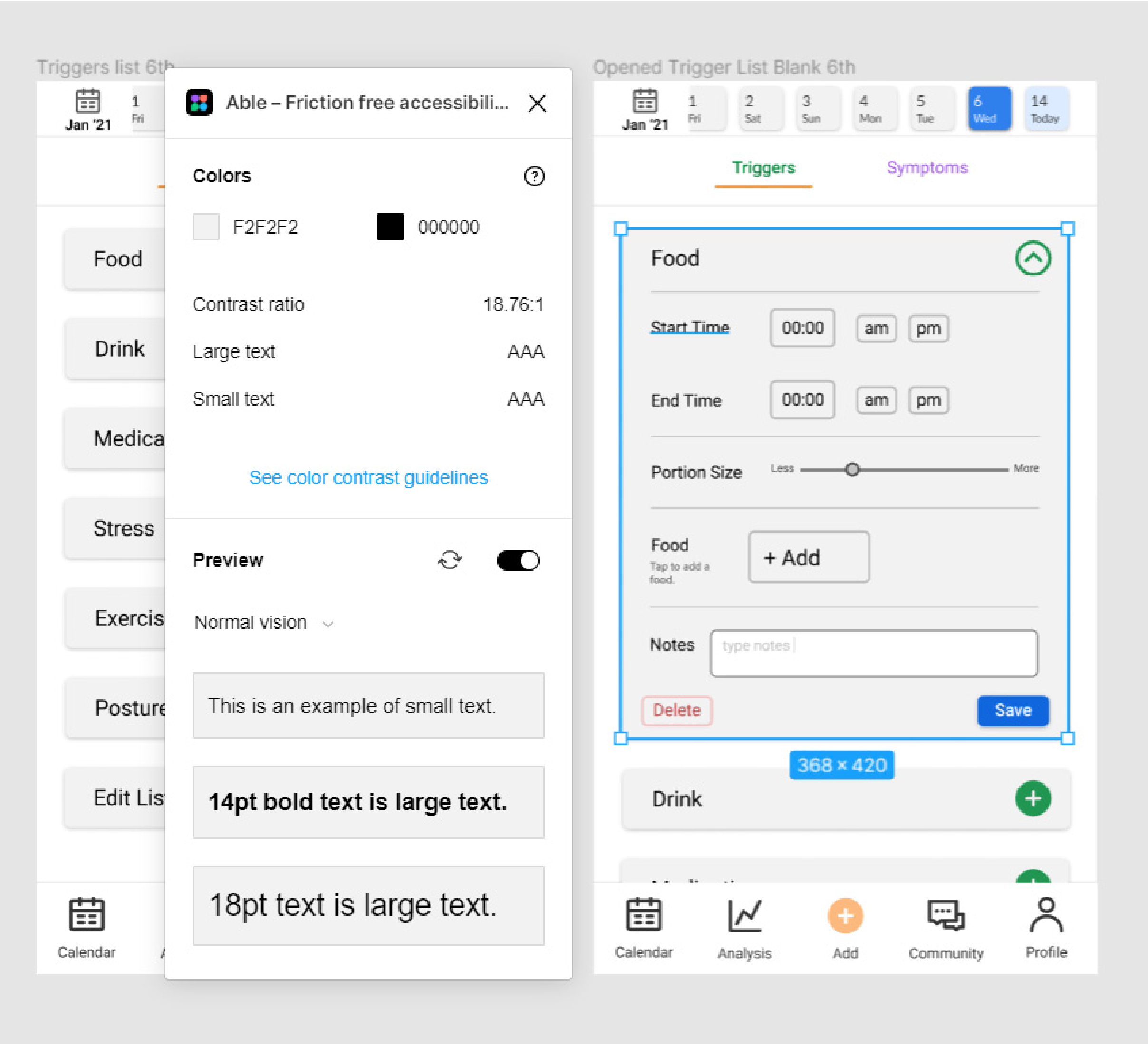

Figure S13. Able Contrast Test.

The profile button leads to a page where users can customize their profile information, change their notification settings, set alarms, connect wearable devices, and adjust app settings. Users may press the edit button to set their profile picture or change their basic demographic information. Pressing designated buttons will lead users to other setting and function pages. The devices feature allows users to sync wearable technology with the app, so that the users can get alarms and notifications about their acid reflux. On this page, users can easily add, delete, and re-order wearable technology devices. An alarms page allows users to set, edit, and delete alarms for mealtimes, water intake, or any other routine. Last, the download data function—which is also available on the analysis page—is available via the settings page because users often look for data-export features under settings. We chose to place this function in two locations, so that users can access it intuitively.

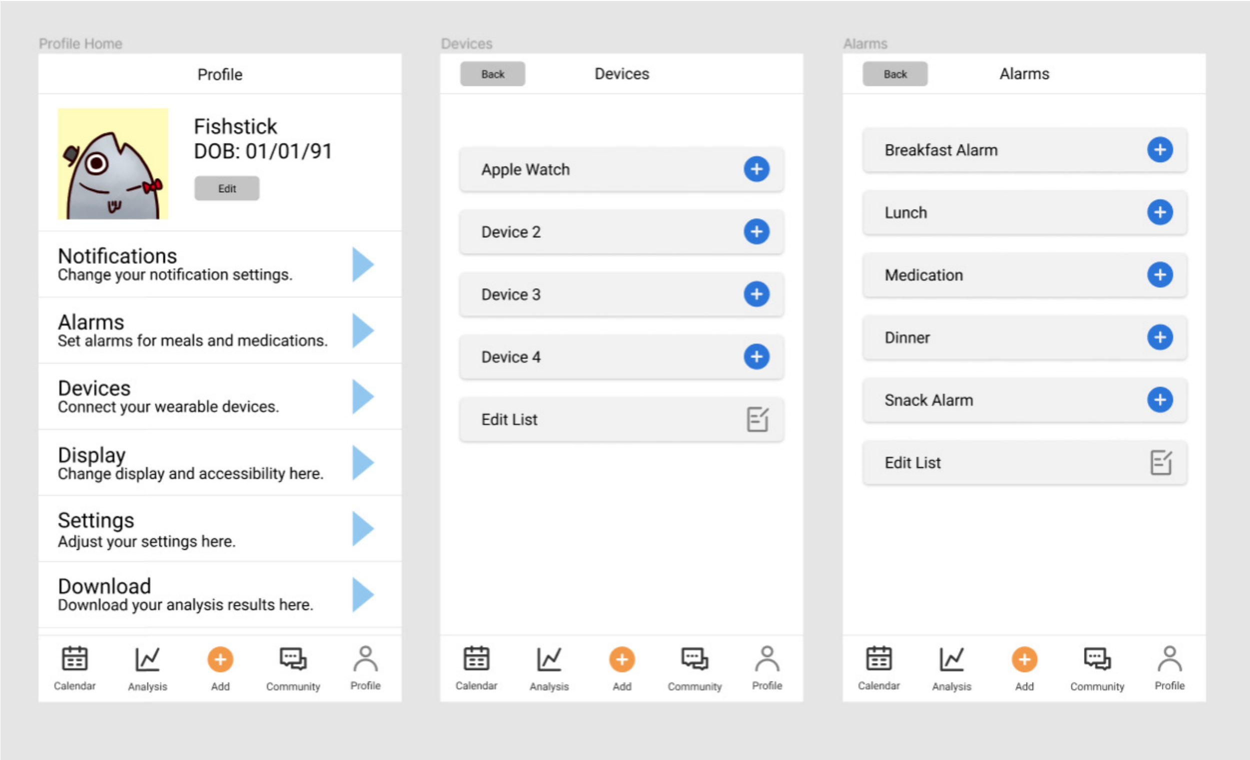

Figure S14. Profile Page.

The final GerdHelper prototype, submitted to the HFES Mobile Health Applications for Consumers Design Competition, is shown in its entirity below and can be accessed online: https://www.figma.com/proto/5FtbZhuQyptP5854g4ydgb/Post-FINAL-Our-Health-App-Sketch-Prototype?node-id=336%3A5371&starting-point-node-id=80%3A237 (Accessed on 10 January 2022)

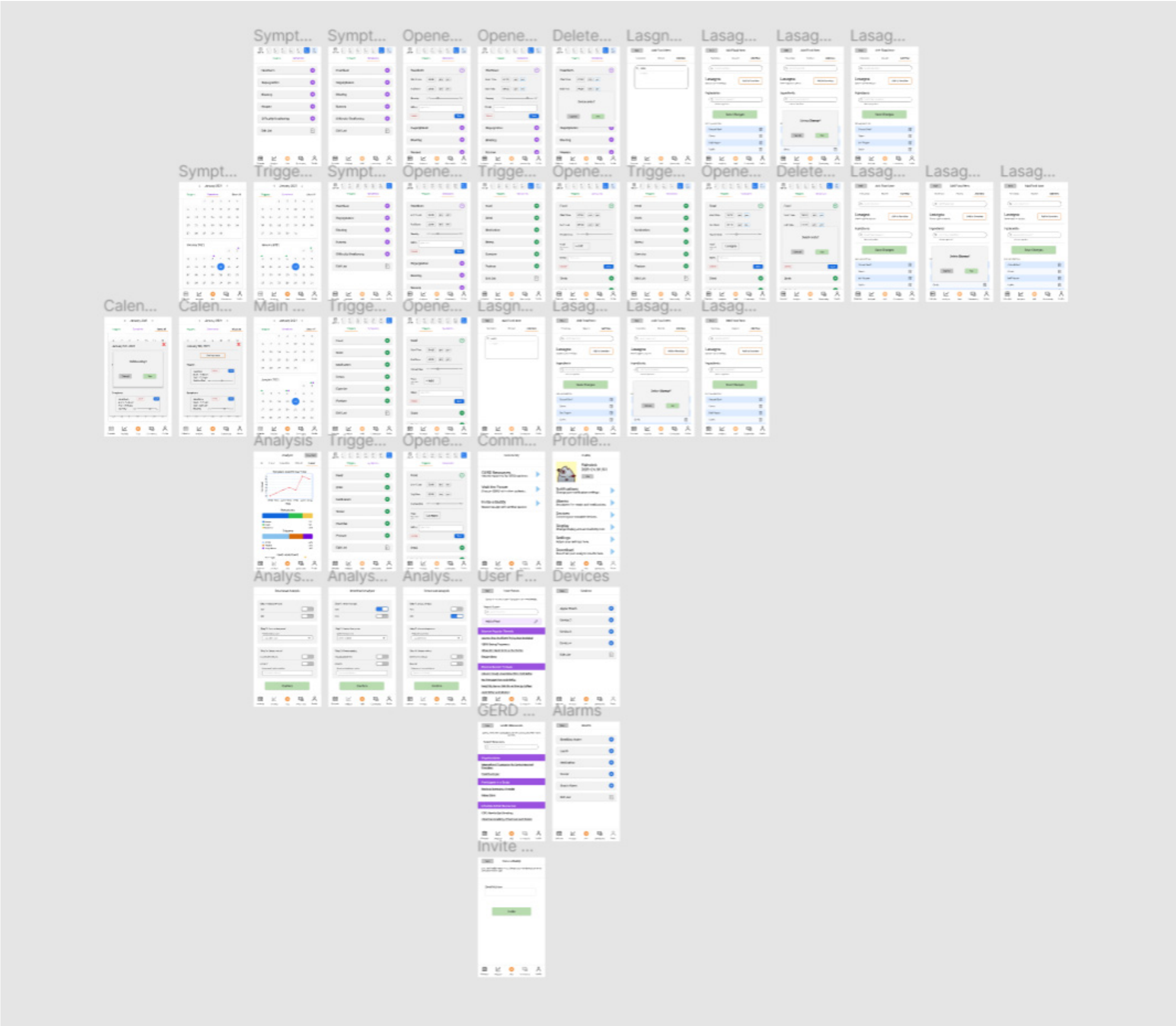

Figure S15. Final Figma Prototype Screens.

The final GerdHelper calendar page by default displays a calendar of the past two months, where a default "Show All" tab highlights the days on which both symptoms and triggers were entered. Additional "Symptoms" and "Triggers" tabs are available in case the user wants to see only one or the other. The calendar view allows users to see days on which they had certain symptoms and triggers over a longer period of time. Symptom and trigger records are differentiated by both color and shape, so that users can quickly determine which information is recorded on which days. When users click on a date on the calendar, a pop-up list shows all of the triggers and symptoms recorded on that day. The pop-up list shows the types of triggers/symptoms, when these triggers/symptoms occurred, and supplemental data such as food portion size and symptom severity. An edit button allows users to easily edit data as needed, and a calendar button at the top left of the screen allows users to return to the calendar at any time. The top-left calendar button's location is analogous to the location of the back button in other sections of the app.

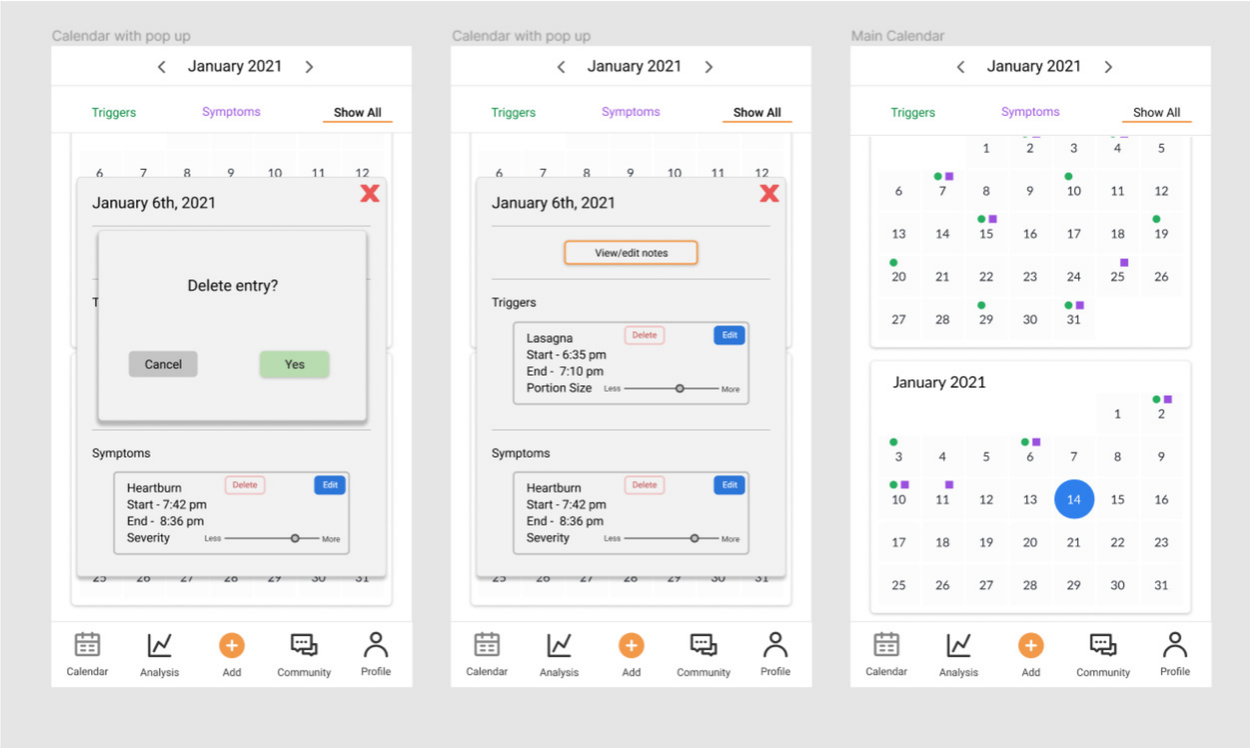

Figure S16. Final Calendar.

The final analysis button leads to a page that sums up the user's recorded data including symptom severity over time, frequent triggers and symptoms, an analysis summary, and a food leaderboard that lists foods to avoid. The app compiles this data by identifying symptom severity, as well as overlap between symptoms and triggers. At the top of the analysis page, the user may toggle among analysis displays for the past week, month, or year. A download button at the top right of the analysis page prompts a pop-up page where users can choose between two file types (.csv and .pdf) and choose to either download the data or share the data via email.

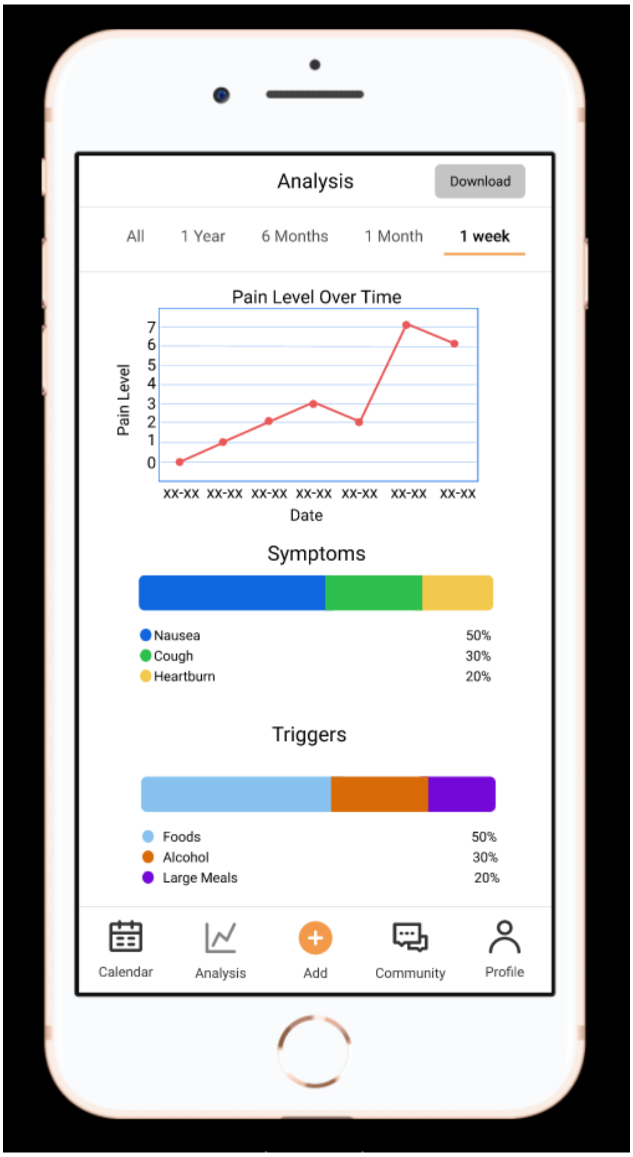

Figure S17. Analysis Page.

The final "add" button leads to a page where users can record their triggers and symptoms. This page features default lists of common acid reflux triggers and symptoms; users have the option to delete, add, or re-order triggers and symptoms using an "Edit List" option. For each trigger users are prompted to add details such as time, intensity of exercise, food portion size, food ingredients, etc. Users may also add notes about their trigger entries. Similarly, when entering symptoms, users may record start and end time, symptom severity, and notes. Default symptoms and triggers were chosen on the basis of user personas and user interviews. After a trigger or symptom gets added, a save confirmation will pop up to provide a visual reassurance that the information the user input has been saved. (We were unable to prototype this feature due to the limitations of Figma.)

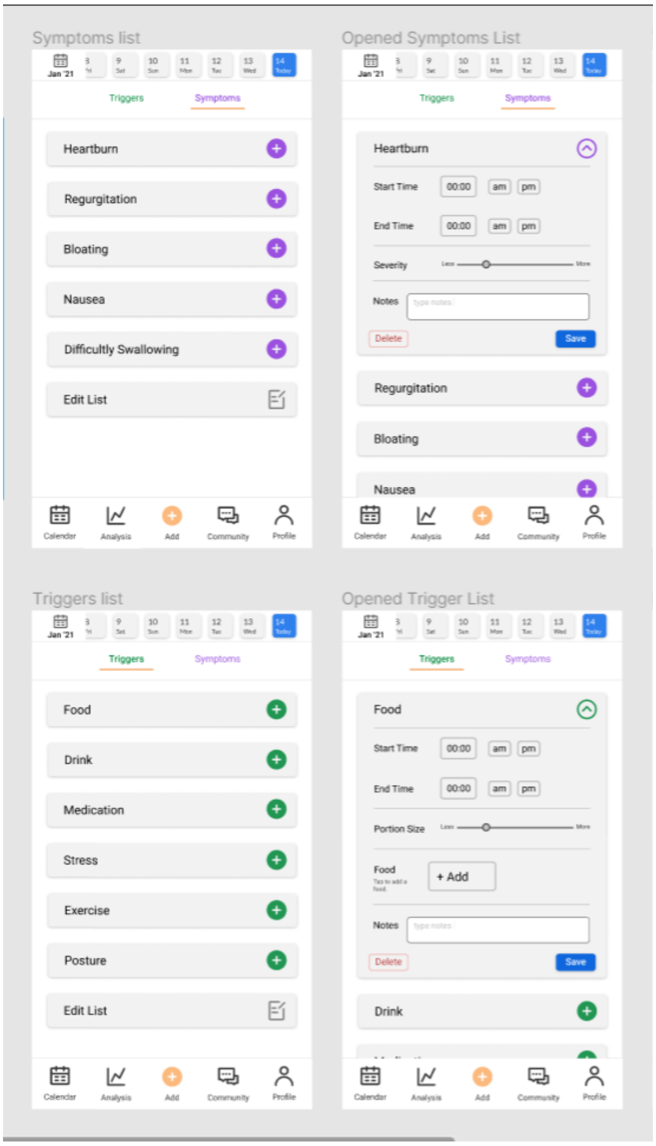

Figure S18. Symptom and Trigger Adding.

The community button leads to a menu page listing three subpages. These three subpages are titled GERD Resources, Visit the Forum, and Invite a Buddy. GERD Resources will include expert-vetted web resources where users can access credible information about GERD treatments, GERD research, and access to healthcare. On the Visit the Forum page, users can post, search, and interact with posts made by other app users. A search bar is located at the top of the forum, followed by popular threads and recently posted threads. Anyone can post on these forums, but experts will get a verified-check symbol next to their posts. All posts will be reviewed by a moderator before publication to prevent offensive or inappropriate content, but users will also have the option to report inappropriate content in the event that it is overlooked by moderators. Finally, the Invite a Buddy page will allow users to invite others to use the app by adding an email address to the text bar and pressing the invite button.

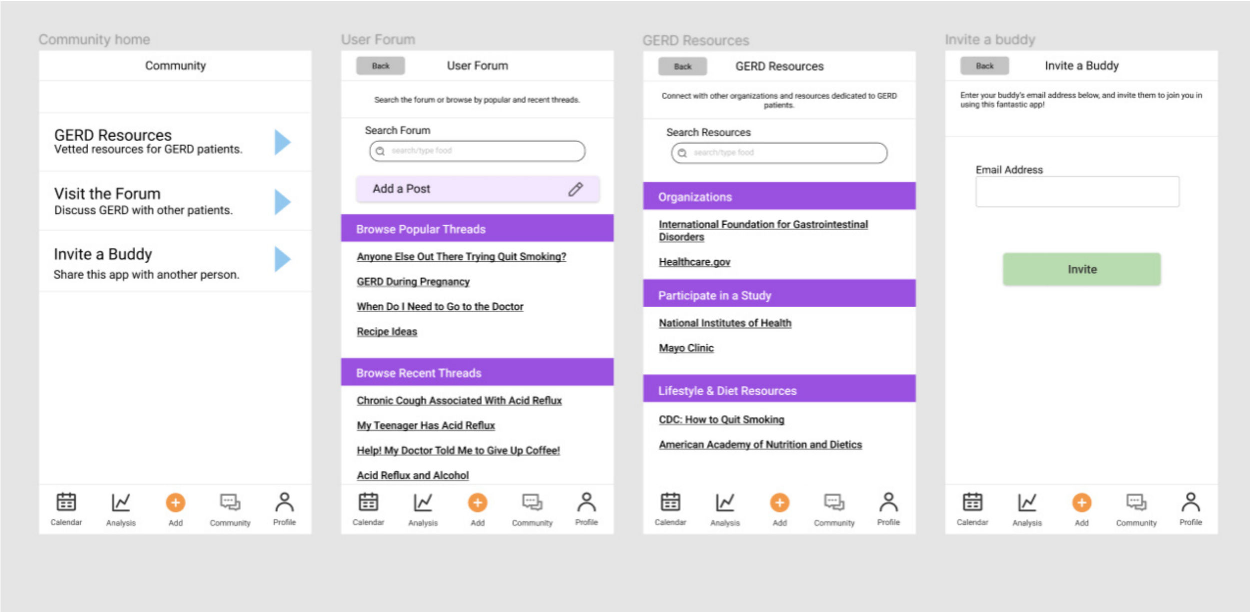

Figure S19. Community Pages.

Supplementary E

To collect feedback on GerdHelper from exeprts in the treatment of GERD, we emailed the following survey to 25 experts, of whom two submitted answers to all questions.

1. What is your expertise in GERD? (Answer Choices: I am a medical practicioner, I am a research scientist, or I am both a medical practitioner and a research scientist)
2. What is your highest level of education? (Answer Choices, of which multiple could be selected: MD, PhD, or Other; note that those who answered other were asked to provide additional information)
3. Briefly describe your experience studying GERD or treating GERD patients
4. Which behaviors can contribute to the treatment of GERD
5. Do you think that a mobile app could be useful in treating GERD, and if so what features would be useful?
6. Briefly describe what informaiton is most important for GERD patients to be aware of and what symptoms and behaviors are most useful for them to keep track of
7. What methods have you seen patients use to successfully track GERD symptoms and related behaviors?
8. Have you seen patients unsuccessfully attempt to track GERD symptoms and related behaviors? Why do you think they were unsuccessful?
9. Have you seen GERD patients successfully use mobile apps to manage their condition, and if so what factors controbuted to their success?
10. What tools do you wish were available to GERD patients?
11. What information related to GERD do you wish ere easier to keep track of (for either you or patientss)?
12. For the remainder of this survey, please refer to the following medium-fidelity prototyps of an app to treat GERD (Accessed online: https://www.figma.com/proto/5FtbZhuQyptP5854g4ydgb/Post-FINAL-Our-Health-App-Sketch-Prototype?node-id=80%3A237&viewport=-1260%2C367%2C0.5&scaling=scale-down&page-id=0%3A1 (Accessed on 10 January 2022)
13. Would you find this app useful for GERD patients? Which parts and why?

14. Would you recommend this app to patients? What, if any, hesitancies would you have in recommending it and why?
15. Aw a practitioner/researcher, would you find any of the data collected by this app useful? Why or why not?
16. Specifically, which information collected by this app could be useful for treating GERD?
17. Which if any features could be added to this app to make it more useful for treating GERD?
